# Bayesian estimation of the inverse Exponential Power distribution for COVID-19 case fatality analysis under SDG 3

**DOI:** 10.1038/s41598-025-27264-7

**Published:** 2025-12-01

**Authors:** Neriman Akdam, Osama Abdulaziz Alamri, Subhankar Dutta, Fatma Çiftci, Neslihan İyit

**Affiliations:** 1 Department of Biostatistics, Faculty of Medicine, Selcuk University, Konya, Turkey; 2 Department of Statistics, Faculty of Science, University of Tabuk, Tabuk, Saudi Arabia; 3 Department of Mathematics, Bioinformatics and Computer Applications, Maulana Azad National Institute of Technology, Bhopal, Madhya Pradesh India; 4 Department of Industrial Engineering, Faculty of Engineering and Natural Sciences, KTO Karatay University, Konya, Turkey; 5 Department of Statistics, Faculty of Science, Selcuk University, Konya, Turkey

**Keywords:** Bayes estimator, Inverse Exponential Power distribution, Lindley’s approximation, Markov Chain Monte Carlo method, Tierney–Kadene’s approximation, Squared-error loss function, Sustainable development goal, COVID-19 pandemic, Case fatality rate, World Health Organization, Organisation for Economic Co-Operation and Development, Statistics, Scientific data

## Abstract

In this study, the maximum likelihood estimators (MLEs) and Bayes estimators for the shape and scale parameters of Inverse Exponential Power (IEP) distribution are derived. As closed-form solutions for the Bayes estimators are not available, approximate estimators are obtained through Lindley’s and Tierney–Kadane’s approximation methods, along with the Markov Chain Monte Carlo (MCMC) method, under the squared-error loss (SEL) function. Also, the approximate Bayes estimates are evaluated against the maximum likelihood estimates based on mean square error (MSE) and bias values using Monte Carlo simulation. In addition, the coverage probabilities of the parametric bootstrap estimates are computed. Finally, real data sets belonging to the COVID-19 Pandemic Case Fatality Rate across World Health Organization (WHO) and Organization fo Economic Co-Operation and Development (OECD) regions data is investigated as an important indicator to achieve United Nations’ Sustainable Development Goal 3 (SDG 3) are employed to display the emprical results associated with both non-bayesian and bayesian estimations of the IEP distribution presented. By offering improved estimation techniques for flexible health indicator distributions, the results contribute to the broader effort of enhancing statistical tools used in global health analytics—particularly in areas such as survival modeling, biomedical reliability, and chronic disease monitoring aligned with SDG 3.

**Supplementary Information:**

The online version contains supplementary material available at 10.1038/s41598-025-27264-7.

## Introduction

In reliability and survival analysis, lifetime distributions are needed to measure the average lifetime of components of a system. Because life sciences, medicine, biology, engineering, actuarial science, and other professions frequently employ lifetime distributions to model and interpret lifetime data. The exponential distribution is one of the most preferred distribution for modeling real lifetime data due to the flexibility property of this distribution. There are many generalizations of exponential distribution for more flexible modeling in the literature such as Gupta and Kundu^1^, Dey and Dey^2^, Oguntunde et al.^3^, Afify et al.^4^, Afify et al.^5^, and Alghamedi et al.^6^.

The exponential power model proposed by Smith et al.^7^ is established by modifying the exponential distribution. The exponential power distribution assumes a bathtub-shaped hazard function as an extreme-value type model^8^. The inverse exponential power (IEP) distribution introduced by Chaudhary et al. (2023) is based on the exponential power distribution to analyze lifetime data in the reliability and survival analysis having both bathtub-shaped and also j-shaped failure rate functions of the parameters Chaudhary et al.^9^.

The IEP distribution with the parameters $\left( {\alpha ,\lambda } \right)$ is shown with ${\text{IEP}}\left( {{\alpha ,}\lambda } \right)$, where $\alpha > 0$ and $\lambda > 0$ are the scale and shape parameters respectively. The probability density function (pdf), cumulative distribution function (cdf), survival function, and hazard function of *X* random variable has exponential power distribution with $\alpha$ and $\lambda$ parameters are as follows;

1 $$f(x;\alpha ,\lambda ) = \frac{\alpha }{\lambda }\left( {\frac{\lambda }{x}} \right)^{\alpha + 1} \exp \left( {\frac{\lambda }{x}} \right)^{\alpha } \exp \left[ {1 - \exp \left( {\frac{\lambda }{x}} \right)^{\alpha } } \right],x > 0$$

2 $$F\left( {x;\alpha ,\lambda } \right) = \exp \left[ {1 - \exp \left( {\frac{\lambda }{x}} \right)^{\alpha } } \right],\,\,x > 0$$

3 $$R(x;\alpha ,\lambda ) = 1 - \exp \left[ {1 - \exp \left( {\frac{\lambda }{x}} \right)^{\alpha } } \right],\,\,x > 0$$

4 $$h\left( {x;\alpha ,\lambda } \right) = \frac{{{\alpha \mathord{\left/ {\vphantom {\alpha \lambda }} \right. \kern-0pt} \lambda }\left( {{\lambda \mathord{\left/ {\vphantom {\lambda x}} \right. \kern-0pt} x}} \right)^{\alpha + 1} \exp \left( {{\lambda \mathord{\left/ {\vphantom {\lambda x}} \right. \kern-0pt} x}} \right)^{\alpha } \exp \left[ {1 - \exp \left( {{\lambda \mathord{\left/ {\vphantom {\lambda x}} \right. \kern-0pt} x}} \right)^{\alpha } } \right]}}{{1 - \exp \left[ {1 - \exp \left( {{\lambda \mathord{\left/ {\vphantom {\lambda x}} \right. \kern-0pt} x}} \right)^{\alpha } } \right]}},\,x > 0$$

**Fig. 1:**
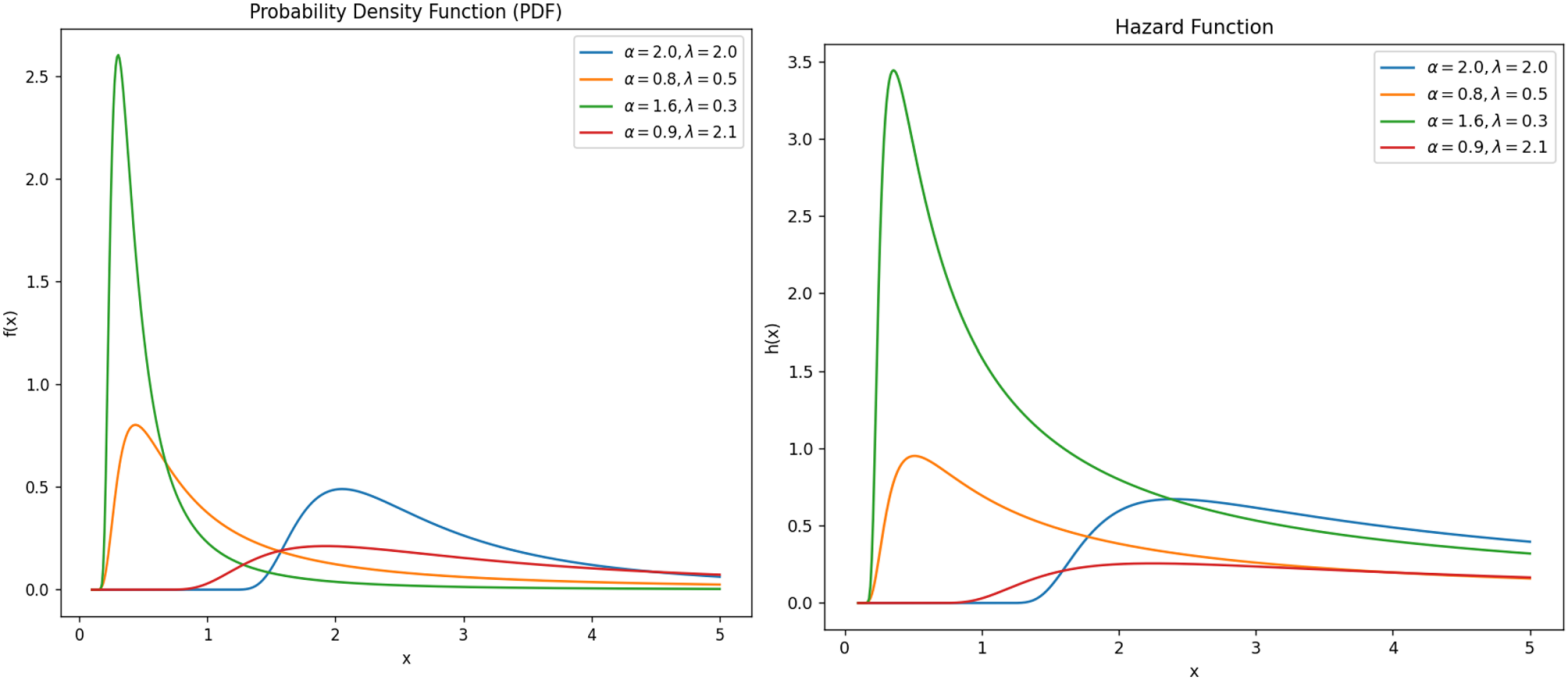
PDF and Hazard function graphs for numerous parameter values.

Figure 1 represents the PDF and hazard function for different values of the parameters of the IEP distribution. In this study, non-Bayesian estimator using maximum likelihood (ML) method and Bayesian estimators using Lindley’s and Tierney–Kadane’s approximations and Monte Carlo Markov Chain (MCMC) method for parameters of the IEP distribution are investigated in details, comparatively in the aspect of bias and mean square error (MSE). There are many studies in the literature on non-Bayesian and Bayesian parameter and also reliability estimates of different distributions under complete and censored samples. For more detailed information see Karadayı et al.^10^, Preda and Panaitescu^11^, Akdam et al.^12^, Sharma et al.^13^, Mashail and Sobhi^14^, Sule and Adegoke^15^, Afify et al.^16^, El-Morshedy et al.^17^, Akdam^18^, Çitci et al.^19^, Akdam^20^, and Zhuang^21^.

Although the IEP distribution was originally introduced by Chaudhary et al. (2023), this study makes several novel contributions to the literature. Specifically, this is the first attempt to derive Bayesian estimators for the shape and scale parameters of the IEP distribution using Lindley’s approximation, Tierney–Kadane expansion, and the Markov Chain Monte Carlo (MCMC) method under the squared-error loss (SEL) function. Furthermore, a comprehensive comparison of these estimators with maximum likelihood estimates (MLEs) using Monte Carlo simulations is provided in terms of mean squared error and bias.

Beyond the theoretical contribution of the IEP distribution, the proposed methodology also bears practical relevance in medical data analysis, especially in modeling deterioration processes, survival times, and event occurrence patterns. Such applications are aligned with the broader objectives of SDG 3, which emphasizes advancing health systems, optimizing clinical outcomes, and supporting innovation in health-related statistical modeling. So finally, the empirical application of the IEP model to the COVID-19 Case Fatality Rate data across World Health Organization (WHO) and Organization fo Economic Co-Operation and Development (OECD) regions offers a novel public health perspective by linking statistical modeling with global development indicators such as SDG 3.

This study presents several strengths in both methodology and application. First, the comprehensive formulation of the IEP distribution provides a solid foundation for the COVID-19 pandemic case fatality rate data modeling. Second, the integration of multiple Bayesian estimation approaches, including Lindley’s approximation, Tierney–Kadane expansion, and Markov Chain Monte Carlo (MCMC) methods, offers a robust comparative framework that is rarely encountered in the literature. Third, the use of the MSE and bias as performance metrics across a wide range of sample sizes ensures a rigorous evaluation of the estimator performance. Furthermore, the empirical application involving real-world COVID-19 Case Fatality Rate (CFR) data from WHO and OECD regions enhances the practical relevance of the model. By linking the statistical modeling to the SDG 3, this study not only addresses methodological innovation but also makes a meaningful contribution to the global health analytics.

The IEP distribution offers greater flexibility than the classical exponential or Weibull models due to its ability to accommodate heavy tails and varying hazard shapes. These properties make it highly suitable for applications involving medical or biological data, where the risk of event occurrence may increase or decrease over time. For instance, the IEP model can be utilized in analyzing survival times of patients, degradation of medical devices, or the progression of chronic diseases—domains where accurate estimation is crucial for public health interventions and resource allocation.

The primary goal of this research is to develop the Bayes estimators (BEs) under square error loss (SEL) functions and compare them with the MLEs in terms of bias and MSE values of the estimates. This article is divided into the following sections: In Sect. “Introduction”, an outline of the IEP distribution is provided. The MLEs and bootstrap confidence intervals for model parameters are derived in Sect. “Maximum Likelihood Estimation (MLE)”. Section “Bayes estimation” deduces the BEs under the SEL function through Lindley’s and Tierney–Kadane’s approximations as well as MCMC method using Monte Carlo simulation. And then the approximate Bayes estimations are contrasted with the ML estimations of the parameters in terms of the MSE and bias values and also the relevalent results are tabulated in Sect. “Simulation study”. Using the COVID-19 Pandemic Case Fatality Rate data, an important indicator for achieving the United Nations’ SDG 3, non-bayesian and bayesian estimates of the IEP distribution are provided at in Sect. “Real data application of the Inverse Exponential Power (IEP) distribution to the COVID-19 pandemic case fatality rate”. Finally, main conclusions to compare these estimators of the IEP distribution are also provided.

## Maximum Likelihood Estimation (MLE)

Let $\left( {X_{1} ,X_{2} , \ldots ,X_{n} } \right)$ denote a random sample taken from the $IEP\left( {{\alpha ,}\lambda } \right)$ distribution with observed values $\left( {x_{1} ,x_{2} , \ldots ,x_{n} } \right)$. Then, the log-likelihood function $L\left( {\alpha ,\lambda } \right) = \log \ell \left( {\alpha ,\lambda } \right)$ can be written as

5 $$L\left( {\alpha ,\lambda \left| {\underline{x} } \right.} \right) = n\log \left( \alpha \right) + n\alpha \log \left( \lambda \right) - \left( {\alpha + 1} \right)\sum\limits_{i = 1}^{n} {\log \left( {x_{i} } \right) + \lambda^{\alpha } \sum\limits_{i = 1}^{n} {x_{i}^{ - \alpha } } + n - \sum\limits_{i = 1}^{n} {\exp \left[ {\left( {\frac{\lambda }{{x_{i} }}} \right)^{\alpha } } \right]} }$$

Taking the partial derivatives of $L\left( {\alpha ,\lambda } \right)$ according to $\alpha$ and $\lambda$ parameters, L_1_ and L_2_ equations are achived;

6 $$L_{1} = \frac{\partial L}{{\partial \alpha }} = \frac{n}{\alpha } + n\log \left( \lambda \right) - \sum\limits_{i = 1}^{n} {\log \left( {x_{i} } \right)} + \sum\limits_{i = 1}^{n} {\left( {\frac{\lambda }{{x_{i} }}} \right)^{\alpha } } \log \left( {\frac{\lambda }{{x_{i} }}} \right)\left[ {1 - \exp \left( {\frac{\lambda }{{x_{i} }}} \right)^{\alpha } } \right] = 0$$

7 $$L_{2} = \frac{\partial L}{{\partial \lambda }} = \frac{n\alpha }{\lambda } + \sum\limits_{i = 1}^{n} {\alpha \lambda^{\alpha - 1} } x_{i}^{\alpha } \left[ {1 - \exp \left( {\frac{\lambda }{{x_{i} }}} \right)^{\alpha } } \right] = 0$$

Since the nonlinear equations can not be expressed in closed form, Eqs. (6) and (7) can be computed by the Newton–Raphson method.

### Asymptotic Confidence Interval (ACI)

Let $\phi = \left( {\alpha ,\lambda } \right)$ be the Fisher information matrix of $\phi$ parameter vector given by

8 $$I\left( \phi \right) = - E\left[ {\begin{array}{*{20}c} {\frac{{\partial^{2} \log \ell }}{{\partial \alpha^{2} }}} & {\frac{{\partial^{2} \log \ell }}{\partial \alpha \partial \lambda }} \\ {\frac{{\partial^{2} \log \ell }}{\partial \alpha \partial \lambda }} & {\frac{{\partial^{2} \log \ell }}{{\partial \lambda^{2} }}} \\ \end{array} } \right]$$

Since the Fisher information matrix $I\left( \phi \right)$ given in Eq. (8) is difficult to compute, the observed Fisher information matrix $I\left( {\hat{\phi }} \right)$ is used approximate to expect Fisher information matix where ${{\varvec{\upphi}}} = \left( {\alpha ,\lambda } \right)$ is the MLEs of the parameters ${\hat{\mathbf{\phi }}} = \left( {\hat{\alpha },\hat{\lambda }} \right)$ as follows^22^;

9 $$I\left( {\hat{\phi }} \right) = \left[ {\begin{array}{*{20}c} { - \frac{{\partial^{2} \log \ell }}{{\partial \alpha^{2} }}} & { - \frac{{\partial^{2} \log \ell }}{\partial \alpha \partial \lambda }} \\ { - \frac{{\partial^{2} \log \ell }}{\partial \alpha \partial \lambda }} & { - \frac{{\partial^{2} \log \ell }}{{\partial \lambda^{2} }}} \\ \end{array} } \right]_{{\phi = \hat{\phi }}}$$

These second order derivatives of the log-likelihood are provided in the supplementary file of the manuscript. The observed variance–covariance matrix for the MLEs $\left( {\hat{\alpha },\hat{\lambda }} \right)$ is the inverse of the observed information matrix as follows;

10 $$I^{ - 1} \left( {\hat{\phi }} \right) = \left[ {\begin{array}{*{20}c} {\hat{V}ar\left( {\hat{\alpha }} \right)} & {\hat{C}ov\left( {\hat{\alpha },\hat{\lambda }} \right)} \\ {\hat{C}ov\left( {\hat{\alpha },\hat{\lambda }} \right)} & {\hat{V}ar\left( {\hat{\lambda }} \right)} \\ \end{array} } \right]$$

$\hat{\phi }$ is approximately bivariately normaly distributed with mean $\phi$ and variance–covariance matrix $I^{ - 1} \left( {\hat{\phi }} \right)$ as $\left[ {\hat{\phi } \sim N\left( {\phi ,I^{ - 1} \left( {\hat{\phi }} \right)} \right)} \right]$ ^17^. Thus, the 100 (1−ρ)% confidence interval for $\alpha \,$ and $\lambda$ can be constructed as $\left( {\hat{\alpha } \mp z_{{\frac{\rho }{2}}} \times \sqrt {Var\left( {\hat{\alpha }} \right)} } \right)$ and $\left( {\hat{\lambda } \mp z_{{\frac{\rho }{2}}} \times \sqrt {Var\left( {\hat{\lambda }} \right)} } \right)$ where $z_{\rho }$ denotes the upper ρ-th quantile of the standard normal distribution.

### Bootstrap confidence ınterval

The percentile bootstrap confidence intervals (P-BCI) method suggested by Efron^23^ are used to obtain confidence intervals for the unknown $\alpha$ and $\lambda$ parameters. By using the P-BCI method, the steps to estimate P-BCI intervals of the parameters are as follows^12^;

**Step 1.** Take MLEs of the $\alpha$ and $\lambda$ parameters be $\hat{\alpha }_{MLE}$ and $\hat{\lambda }_{MLE}$, respectively.

**Step 2.** To generate the bootstrap samples, using the $\hat{\alpha }_{MLE}$ and $\hat{\lambda }_{MLE}$, compute the bootstrap estimate of the $\alpha$ and $\lambda$ parameters as $\hat{\alpha }^{*}_{MLE}$ and $\hat{\lambda }^{*}_{MLE}$, respectively.

**Step 3.** Repeat Step 2 NBoot times.

**Step 4.** Take $T^{*} (x) = P\left( {\hat{\alpha }^{*} \le x} \right)$ as the cdf of $\hat{\alpha }^{*}$. Define $\hat{\alpha }^{*} = T^{* - 1} \left( x \right)$ for given $x$. The approximate bootstrap 100(1-α)% confidence interval for $\alpha$ is defined as $\left( {\hat{\alpha }_{{\frac{\gamma }{2}}}^{*} ,\hat{\alpha }_{{1 - \frac{\gamma }{2}}}^{*} } \right)$. Similarly, 100(1-α)% confidence interval for $\lambda$ is defined as $\left( {\hat{\lambda }_{{\frac{\gamma }{2}}}^{*} ,\hat{\lambda }_{{1 - \frac{\gamma }{2}}}^{*} } \right)$.

## Bayes estimation

Assuming independent priors $Gamma\left( {\gamma_{1} ,\delta_{1} } \right)$ and $Gamma\left( {\gamma_{2} ,\delta_{2} } \right)$ densities for the $\alpha$ and $\lambda$ parameters of ${\text{IEP}}\left( {{\alpha ,}\lambda } \right)$ distribution, respectively. In this instance, the joint prior distribution of $\alpha$ and $\lambda$ can be expressed as,

11 $$\pi \left( {\alpha ,\lambda } \right) = \alpha^{{\gamma_{1} - 1}} \delta_{1}^{{\gamma_{1} }} \lambda^{{\gamma_{2} - 1}} \delta_{2}^{{\gamma_{2} }} \exp \left( { - \delta_{1} \alpha } \right)\exp \left( { - \delta_{2} \lambda } \right)\Gamma^{ - 1} \left( {\gamma_{1} } \right)\Gamma^{ - 1} \left( {\gamma_{2} } \right)\,\,\,\,\,\gamma_{i} ,\delta_{i} ,\alpha ,\lambda > 0\,,i = 1,2$$

From (11), the log of prior density function is given by,

12 $$\begin{aligned} \rho \left( {\alpha ,\lambda } \right) = & \left( {\gamma_{1} - 1} \right)\log \left( \alpha \right) + \left( {\gamma_{2} - 1} \right)\log \left( \lambda \right) - \delta_{1} \alpha - \delta_{2} \lambda \\ & \; + \gamma_{1} \log \left( {\delta_{1} } \right) + \gamma_{2} \log \left( {\delta_{2} } \right) - \log \left[ {\Gamma \left( {\gamma_{1} } \right)} \right] - \log \left[ {\Gamma \left( {\gamma_{2} } \right)} \right] \\ \end{aligned}$$

By using $L\left( {\alpha ,\lambda } \right)$ and $\pi \left( {\alpha ,\lambda } \right)$, the joint posterior density function of $\alpha$ and $\lambda$ parameters can be written as

13 $$P\left( {\alpha ,\lambda \left| {\rm X} \right.} \right) \propto \frac{{\alpha^{{n + \gamma_{1} - 1}} \lambda^{{ - n + \gamma_{2} - 1}} \exp \left( { - \delta_{1} \alpha } \right)\exp \left( { - \delta_{2} \lambda } \right)\exp \left[ {\sum\limits_{i = 1}^{n} {\left( {\frac{\lambda }{{x_{i} }}} \right)^{\alpha } } } \right]\exp \left[ {\sum\limits_{i = 1}^{n} {1 - \exp \left( {\frac{\lambda }{{x_{i} }}} \right)^{\alpha } } } \right]}}{{\int\limits_{0}^{\infty } {\int\limits_{0}^{\infty } {\alpha^{{n + \gamma_{1} - 1}} \lambda^{{ - n + \gamma_{2} - 1}} \exp \left[ {\sum\limits_{i = 1}^{n} {\left( {\frac{\lambda }{{x_{i} }}} \right)^{\alpha } } } \right]\exp \left[ {\sum\limits_{i = 1}^{n} {1 - \exp \left( {\frac{\lambda }{{x_{i} }}} \right)^{\alpha } } } \right]\,d\alpha d\lambda } } }}$$

Therefore, the Bayes estimate of any function of $\alpha$ and $\lambda$, say $u\left( {\alpha ,\lambda } \right)$, under a SEL function can be expressed as

14 $$\hat{u}_{B} \left( {\alpha ,\lambda } \right) = E\left[ {u\left( {\alpha ,\lambda } \right)} \right] = \frac{{\int\limits_{0}^{\infty } {\int\limits_{0}^{\infty } {u\left( {\alpha ,\lambda } \right)e^{{\left[ {L\left( {\alpha ,\lambda } \right) + \rho \left( {\alpha ,\lambda } \right)} \right]}} \,\,d\alpha d\lambda } } }}{{\int\limits_{0}^{\infty } {\int\limits_{0}^{\infty } {e^{{\left[ {L\left( {\alpha ,\lambda } \right) + \rho \left( {\alpha ,\lambda } \right)} \right]}} d\alpha d\lambda } } }}$$

Since BEs can not be assessed in explicit form using (14), Lindley’s, Tierney–Kadane’s approximation and MCMC method under the SEL function are used to calculate these BEs for the model parameters.

### Lindley’s approximation

In this subsection, an approximation proposed by Lindley^24^ is used to assess the BEs. Lindley approximation is applied to approximately calculate the ratio of two analytically insoluble integrals given in Eq. (14). The formulas of the Lindley approximation for $\alpha$ and $\lambda$ parameters given as follows;

15 $$\begin{gathered} u_{BL} \left( {\hat{\alpha },\hat{\lambda }} \right) = E\left[ {u\left( {\alpha ,\lambda } \right)\left| X \right.} \right] \approx \hfill \\ \left[ {u\left( {\hat{\alpha },\hat{\lambda }} \right) + \frac{1}{2}\sum\limits_{i = 1}^{2} {\sum\limits_{j = 1}^{2} {\left( {u_{ij} + 2u_{i} \rho_{j} } \right)\sigma_{ij} } } + \frac{1}{2}\sum\limits_{i = 1}^{2} {\sum\limits_{j = 1}^{2} {\sum\limits_{k = 1}^{2} {\sum\limits_{l = 1}^{2} {L_{ijk} \sigma_{ij} \sigma_{kl} u_{l} } } } } } \right] \hfill \\ \end{gathered}$$

where $\hat{\alpha }$ and $\hat{\lambda }$ are the MLEs of $\alpha$ and $\lambda$, respectively, and $u_{i} ,\,i = 1,2$ are the unary partial derivatives and $u_{ij} ,\,i,j = 1,2$ are the binary partial derivatives of $\text{u}\left(\uplambda ,\text{c},{\uptheta }_{1},{\uptheta }_{2}\right)$ $u\left( {\alpha ,\lambda } \right)$ with respect to $\alpha$ and $\lambda$ parameters, respectively.$L_{ij} \,,\,i,j = 1,2$ are the binary partial derivatives and ${\text{l}}_{\text{ijk}},\text{i},\text{j},\text{k}=\text{1,2},\text{3,4}$ $L_{ijk} \,,\,i,j,k = 1,2$ are the trinary partial derivatives of log-likelihood function $L\left( {\alpha ,\lambda } \right)$ with respect to $\alpha$ and $\lambda$ parameters, respectively, and $\left[ { - L_{ij} } \right]^{ - 1} = \left[ {\sigma_{ij} } \right]\,, \, i,j = 1,2$ . $\sigma_{ij}$ is the (*i*, *j*)-th element of the matrix $\left[ {\sigma_{ij} } \right]$. From Eq. (9), we get.

$$\rho_{1} = \frac{{\partial \rho \left( {\alpha ,\lambda } \right)}}{\partial \alpha } = \frac{{\gamma_{1} - 1}}{\alpha } - \delta_{1}$$,$$\rho_{2} = \frac{{\partial \rho \left( {\alpha ,\lambda } \right)}}{\partial \lambda } = \frac{{\gamma_{2} - 1}}{\lambda } - \delta_{2}$$.

and then, we derived the values of $L_{ij}$ for *i*, *j* = 1,2 and $L_{ijk}$ for *i*, *j,k* = 1,2 given in Appendix as A1–A10.

The approximate BEs for the parameter of $IEP\left( {\alpha ,\lambda } \right)$ under the SEL function are given by;

16 $$\hat{\alpha }_{BL} = \hat{\alpha }_{MLE} + \frac{1}{2}\sum\limits_{i = 1}^{2} {\sum\limits_{j = 1}^{2} {\left( {u_{ij} + 2u_{i} \rho_{j} } \right)\sigma_{ij} } } + \frac{1}{2}\sum\limits_{i = 1}^{2} {\sum\limits_{j = 1}^{2} {\sum\limits_{k = 1}^{2} {\sum\limits_{l = 1}^{2} {L_{ijk} \sigma_{ij} \sigma_{kl} u_{l} } } } } ,$$

17 $$\hat{\lambda }_{BL} = \hat{\lambda }_{MLE} + \frac{1}{2}\sum\limits_{i = 1}^{2} {\sum\limits_{j = 1}^{2} {\left( {u_{ij} + 2u_{i} \rho_{j} } \right)\sigma_{ij} } } + \frac{1}{2}\sum\limits_{i = 1}^{2} {\sum\limits_{j = 1}^{2} {\sum\limits_{k = 1}^{2} {\sum\limits_{l = 1}^{2} {L_{ijk} \sigma_{ij} \sigma_{kl} u_{l} } } } } ,$$

respectively.

### Tierney–Kadane’s approximation

In this subsection, an approximation suggested by Tierney and Kadane^25^ is used to obtain the BEs. This approximation is as an alternative to Lindley’s approximation and can be defined as shown bellow;

$$\eta (\alpha ,\lambda ) = \frac{1}{n}\left[ {L(\alpha ,\lambda ) + \rho (\alpha ,\lambda )} \right]$$

$$\eta^{*} (\alpha ,\lambda ) = \frac{1}{n}\ln u(\alpha ,\lambda ) + \eta (\alpha ,\lambda )$$

where $L\left( {\alpha ,\lambda } \right)$ denote the log-likelihood function and $\rho \left( {\alpha ,\lambda } \right)$ denote the log of the joint prior density. Thus, by means of Tierney–Kadane’s aproximation Eq. (11) can be written as;

18 $$\begin{aligned} \hat{u}_{BTK} (\alpha ,\lambda ) = & E\left[ {u(\alpha ,\lambda )} \right] = \frac{{\int {e^{{n\eta^{*} (\alpha ,\lambda )}} } d(\alpha ,\lambda )}}{{\int {e^{n\eta (\alpha ,\lambda )} d(\alpha ,\lambda )} }} \\ \approx & \left[ {\frac{{\det \Sigma^{*} }}{\det \Sigma }} \right]^{{{1 \mathord{\left/ {\vphantom {1 2}} \right. \kern-0pt} 2}}} \exp \left\{ {n\left[ {\eta^{*} \left( {\hat{\alpha }^{*} ,\hat{\lambda }^{*} } \right) - \eta \left( {\hat{\alpha },\hat{\lambda }} \right)} \right]} \right\} \\ \end{aligned}$$

where $\left( {\hat{\alpha }^{*} ,\hat{\lambda }^{*} } \right)$ and $\left( {\hat{\alpha },\hat{\lambda }} \right)$ maximize $\eta^{*} \left( {\alpha ,\lambda } \right)$ and $\eta \left( {\alpha ,\lambda } \right)$, respectively. $\Sigma^{*}$ and $\Sigma$ are minus the inverse Hessians of $\eta^{*} \left( {\alpha ,\lambda } \right)$ and $\eta \left( {\alpha ,\lambda } \right)$ at $\left( {\hat{\alpha }^{*} ,\hat{\lambda }^{*} } \right)$ and $\left( {\hat{\alpha },\hat{\lambda }} \right)$.

By means of Tierney–Kadane’s method, BEs of model parameters of $IEP\left( {\alpha ,\lambda } \right)$ under the SEL function are achived, as shown bellow:

$$\eta_{\alpha }^{*} \left( {\alpha ,\lambda } \right) = \frac{\log \left( \alpha \right)}{n} + \eta \left( {\alpha ,\lambda } \right)$$ and $$\Sigma_{\alpha }^{*} = \left[ {\begin{array}{*{20}c} { - \frac{{\partial^{2} \eta_{\alpha }^{*} \left( {\alpha ,\lambda } \right)}}{{\partial \alpha^{2} }}} & { - \frac{{\partial^{2} \eta_{\alpha }^{*} \left( {\alpha ,\lambda } \right)}}{\partial \alpha \partial \lambda }} \\ { - \frac{{\partial^{2} \eta_{\alpha }^{*} \left( {\alpha ,\lambda } \right)}}{\partial \alpha \partial \lambda }} & { - \frac{{\partial^{2} \eta_{\alpha }^{*} \left( {\alpha ,\lambda } \right)}}{{\partial \lambda^{2} }}} \\ \end{array} } \right]^{ - 1}$$

19 $$\hat{\alpha }_{BTK} = \left[ {\frac{{\det \Sigma_{\alpha }^{*} }}{\det \Sigma }} \right]^{{{1 \mathord{\left/ {\vphantom {1 2}} \right. \kern-0pt} 2}}} \exp \left\{ {n\left[ {\eta_{\alpha }^{*} \left( {\hat{\alpha }^{*} ,\hat{\lambda }^{*} } \right) - \eta \left( {\hat{\alpha },\hat{\lambda }} \right)} \right]} \right\}$$

and.

$$\eta_{\lambda }^{*} \left( {\alpha ,\lambda } \right) = \frac{\log \left( \lambda \right)}{n} + \eta \left( {\alpha ,\lambda } \right)$$ and $$\Sigma_{\lambda }^{*} = \left[ {\begin{array}{*{20}c} { - \frac{{\partial^{2} \eta_{\lambda }^{*} \left( {\alpha ,\lambda } \right)}}{{\partial \alpha^{2} }}} & { - \frac{{\partial^{2} \eta_{\lambda }^{*} \left( {\alpha ,\lambda } \right)}}{\partial \alpha \partial \lambda }} \\ { - \frac{{\partial^{2} \eta_{\lambda }^{*} \left( {\alpha ,\lambda } \right)}}{\partial \alpha \partial \lambda }} & { - \frac{{\partial^{2} \eta_{\lambda }^{*} \left( {\alpha ,\lambda } \right)}}{{\partial \lambda^{2} }}} \\ \end{array} } \right]^{ - 1}$$

20 $$\hat{\lambda }_{BTK} = \left[ {\frac{{\det \Sigma_{\lambda }^{*} }}{\det \Sigma }} \right]^{{{1 \mathord{\left/ {\vphantom {1 2}} \right. \kern-0pt} 2}}} \exp \left\{ {n\left[ {\eta_{\lambda }^{*} \left( {\hat{\alpha }^{*} ,\hat{\lambda }^{*} } \right) - \eta \left( {\hat{\alpha },\hat{\lambda }} \right)} \right]} \right\}$$

### Markov Chain Monte Carlo (MCMC)

Markov Chain Monte Carlo (MCMC) is a method used in Bayes estimation. One of the methods for the MCMC approach is the Metropolis–Hastings (M–H) algorithm. The M–H algorithm is produced by Metropolis et al.^26^. In this study, the M–H algorithm is used to achive the BEs of $\alpha$ and $\lambda$ has been employed using *rwmetrop* function in R programme under LearnBayes library (Albert and Albert^27^). For more information regarding the steps of the random-walk M-H algorithm, please refer to Junnumtuam. et al.^28^.

## Simulation study

By means of Monte Carlo simulation study for different sample sizes (*n*) in terms of the MSE and bias the performances of the approximate BEs obtained using Lindley aproximation, Tierney–Kadane aproximation and MCMC method under the SEL function for $\alpha$ and $\lambda$ of the IEP distribution are compared with those of the MLE. Informative priors for $a_{1} = 1,\;b_{1} = 2,\;a_{2} = 2,\;b_{2} = 1$ are used while computing the approximate Bayes estimates. MSE for the estimate of $\alpha$ and $\lambda$ parameters can be calculated with $MSE = \frac{1}{10000}\sum\limits_{i = 1}^{10000} {\left( {\left( {\hat{\alpha }_{i} ,\hat{\lambda }_{i} } \right) - \left( {\alpha ,\lambda } \right)} \right)^{2} }$, where $\left( {\hat{\alpha },\hat{\lambda }} \right)$ is MLE or approximate Bayes estimation. $\alpha \,$ and $\lambda$ is generated from Gamma distribution with parameter $(a_{1} ,b_{1} )$ and $(a_{2} ,b_{2} )$ respectively. All the calculations are based on 10,000 repetitions. Finally, the MSE and bias values are tabulated in Tables 1, 2, 3 and 4.

**Table 1:** ML, appoximate Bayes and MCMC method estimates, MSE and bias values for α = 2 and λ = 2.

*n*	Estımation methods	$\alpha$			$\lambda$		
		Average	Bias	MSE	Average	Bias	MSE
10	MLE	2.3773	0.5904	0.7148	2.0768	0.1776	0.0573
	LINDLEY	2.0572	0.3789	0.2332	1.9805	0.1633	0.0431
	TIERNEY–KADANE	1.6908	0.4071	0.2282	2.0255	0.1620	0.0450
	MCMC	1.6922	0.4105	0.2328	2.0290	0.1629	0.0459
20	MLE	2.1721	0.3580	0.2306	2.0352	0.1162	0.0227
	LINDLEY	2.0535	0.3010	0.1515	1.9871	0.1106	0.0194
	TIERNEY–KADANE	1.8507	0.2940	0.1262	2.0202	0.1124	0.0208
	MCMC	1.8487	0.2921	0.1260	2.0197	0.1145	0.0217
30	MLE	2.1070	0.2695	0.1255	2.0243	0.0930	0.0144
	LINDLEY	2.0350	0.2419	0.0966	1.9921	0.0899	0.0128
	TIERNEY–KADANE	1.8991	0.2376	0.0851	2.0166	0.0911	0.0137
	MCMC	1.9012	0.2407	0.0878	2.0164	0.0928	0.0141
40	MLE	2.0817	0.2300	0.0892	2.0175	0.0798	0.0104
	LINDLEY	2.0294	0.2123	0.0735	1.9934	0.0776	0.0096
	TIERNEY–KADANE	1.9271	0.2078	0.0659	2.0125	0.0786	0.0101
	MCMC	1.9230	0.2089	0.0660	2.0126	0.0793	0.0102
50	MLE	2.0655	0.2014	0.0676	2.0146	0.0718	0.0083
	LINDLEY	2.0246	0.1890	0.0579	1.9953	0.0701	0.0079
	TIERNEY–KADANE	1.9428	0.1855	0.0528	2.0110	0.0710	0.0080
	MCMC	1.9400	0.1884	0.0547	2.0106	0.0708	0.0081
70	MLE	2.0448	0.1661	0.0456	2.0095	0.0597	0.0057
	LINDLEY	2.0164	0.1592	0.0410	1.9957	0.0587	0.0055
	TIERNEY–KADANE	1.9582	0.1578	0.0384	2.0074	0.0592	0.0056
	MCMC	1.9596	0.1594	0.0396	2.0064	0.0594	0.0056
100	MLE	2.0317	0.1374	0.0306	2.0078	0.0499	0.0040
	LINDLEY	2.0121	0.1333	0.0284	1.9981	0.0493	0.0038
	TIERNEY–KADANE	1.9713	0.1321	0.0271	2.0064	0.0496	0.0039
	MCMC	1.9720	0.1340	0.0277	2.0055	0.0495	0.0040
150	MLE	2.0213	0.1117	0.0199	2.0043	0.0400	0.0025
	LINDLEY	2.0084	0.1094	0.0190	1.9978	0.0397	0.0025
	TIERNEY–KADANE	1.9813	0.1083	0.0184	2.0035	0.0399	0.0025
	MCMC	1.9799	0.1087	0.0185	2.0039	0.0409	0.0027
200	MLE	2.0154	0.0958	0.0145	2.0033	0.0347	0.0019
	LINDLEY	2.0058	0.0943	0.0140	1.9985	0.0345	0.0019
	TIERNEY–KADANE	1.9855	0.0939	0.0137	2.0027	0.0346	0.0019
	MCMC	1.9852	0.0943	0.0138	2.0028	0.0348	0.0019
350	MLE	2.0096	0.0728	0.0084	2.0017	0.0266	0.0011
	LINDLEY	2.0041	0.0722	0.0082	1.9989	0.0265	0.0012
	TIERNEY–KADANE	1.9925	0.0720	0.0081	2.0014	0.0266	0.0011
	MCMC	1.9926	0.0717	0.0081	2.0017	0.0266	0.0011
500	MLE	2.0065	0.0606	0.0058	2.0015	0.0218	0.0008
	LINDLEY	2.0027	0.0601	0.0056	1.9996	0.0217	0.0007
	TIERNEY–KADANE	1.9946	0.0600	0.0056	2.0013	0.0218	0.0007
	MCMC	1.9949	0.0605	0.0057	2.0011	0.0223	0.0008

**Table 2:** ML, appoximate Bayes and MCMC method estimates, MSE and bias values, for $\alpha = 0.8$ and $\lambda = 0.5$

*n*	Estımation methods	$\alpha$			$\lambda$		
		Average	Bias	MSE	Average	Bias	MSE
10	MLE	0.9522	0.2396	0.1188	0.5618	0.1214	0.0316
	LINDLEY	0.9213	0.2180	0.0917	0.5309	0.1040	0.0207
	TIERNEY–KADANE	0.8089	0.1697	0.0495	0.6055	0.1416	0.0413
	MCMC	0.8146	0.1716	0.0508	0.6068	0.1416	0.0407
20	MLE	0.8678	0.1407	0.0362	0.5276	0.0759	0.0106
	LINDLEY	0.8552	0.1352	0.0327	0.5145	0.0707	0.0088
	TIERNEY–KADANE	0.8041	0.1200	0.0239	0.5468	0.0823	0.0126
	MCMC	0.8041	0.1218	0.0247	0.5486	0.0838	0.0130
30	MLE	0.8428	0.1090	0.0203	0.5187	0.0599	0.0062
	LINDLEY	0.8344	0.1063	0.0190	0.5103	0.0572	0.0054
	TIERNEY–KADANE	0.8017	0.0981	0.0155	0.5310	0.0634	0.0071
	MCMC	0.8028	0.0982	0.0155	0.5308	0.0640	0.0073
40	MLE	0.8321	0.0907	0.0140	0.5127	0.0510	0.0044
	LINDLEY	0.8257	0.0888	0.0133	0.5066	0.0493	0.0040
	TIERNEY–KADANE	0.8017	0.0837	0.0114	0.5216	0.0531	0.0048
	MCMC	0.8020	0.0861	0.0120	0.5225	0.0540	0.0051
50	MLE	0.8258	0.0809	0.0110	0.5111	0.0455	0.0035
	LINDLEY	0.8207	0.0796	0.0105	0.5062	0.0443	0.0032
	TIERNEY–KADANE	0.8016	0.0759	0.0093	0.5181	0.0470	0.0037
	MCMC	0.8016	0.0761	0.0093	0.5181	0.0473	0.0038
70	MLE	0.8189	0.0671	0.0074	0.5072	0.0374	0.0023
	LINDLEY	0.8152	0.0662	0.0072	0.5038	0.0367	0.0021
	TIERNEY–KADANE	0.8017	0.0639	0.0065	0.5121	0.0383	0.0025
	MCMC	0.8001	0.0652	0.0068	0.5135	0.0391	0.0026
100	MLE	0.8121	0.0551	0.0049	0.5047	0.0312	0.0012
	LINDLEY	0.8095	0.0547	0.0048	0.5023	0.0309	0.0015
	TIERNEY–KADANE	0.8002	0.0534	0.0045	0.5081	0.0317	0.0016
	MCMC	0.8011	0.0537	0.0046	0.5087	0.0320	0.0017
150	MLE	0.8088	0.0449	0.0032	0.5039	0.0251	0.0010
	LINDLEY	0.8070	0.0446	0.0031	0.5023	0.0249	0.0010
	TIERNEY–KADANE	0.8009	0.0439	0.0030	0.5061	0.0254	0.0011
	MCMC	0.8007	0.0437	0.0030	0.5053	0.0258	0.0011
200	MLE	0.8062	0.0387	0.0024	0.5023	0.0219	0.0008
	LINDLEY	0.8049	0.0386	0.0024	0.5011	0.0218	0.0008
	TIERNEY–KADANE	0.8003	0.0382	0.0023	0.5040	0.0221	0.0008
	MCMC	0.8001	0.0378	0.0023	0.5040	0.0222	0.0008
350	MLE	0.8037	0.0288	0.0013	0.5016	0.0166	0.0004
	LINDLEY	0.8029	0.0287	0.0013	0.5009	0.0165	0.0004
	TIERNEY–KADANE	0.8003	0.0285	0.0013	0.5025	0.0167	0.0004
	MCMC	0.8001	0.0285	0.0013	0.5028	0.0165	0.0004
500	MLE	0.8027	0.0242	0.0009	0.5009	0.0138	0.0003
	LINDLEY	0.8021	0.0242	0.0009	0.5005	0.0138	0.0003
	TIERNEY–KADANE	0.8003	0.0240	0.0009	0.5015	0.0138	0.0003
	MCMC	0.8003	0.0242	0.0009	0.5018	0.0139	0.0003

**Table 3:** ML, appoximate Bayes and MCMC method estimates, MSE and bias values, for $\alpha = 1.6$ and $\lambda = 0.3$

*n*	Estimation methods	$\alpha$			$\lambda$		
		Average	Bias	MSE	Average	Bias	MSE
10	MLE	1.9054	0.4780	0.4657	0.3144	0.0333	0.0020
	LINDLEY	1.7135	0.3421	0.2029	0.3086	0.0317	0.0018
	TIERNEY–KADANE	1.4322	0.3132	0.1415	0.3202	0.0357	0.0025
	MCMC	1.4382	0.3142	0.1437	0.3217	0.0369	0.0025
20	MLE	1.7356	0.2814	0.1447	0.3069	0.0220	0.0008
	LINDLEY	1.6648	0.2455	0.1042	0.3040	0.0214	0.0007
	TIERNEY–KADANE	1.5183	0.2307	0.0806	0.3094	0.0227	0.0009
	MCMC	1.5283	0.2337	0.0846	0.3095	0.0228	0.0009
30	MLE	1.6910	0.2216	0.0851	0.3047	0.0176	0.0005
	LINDLEY	1.6472	0.2033	0.0690	0.3028	0.0172	0.0005
	TIERNEY–KADANE	1.5494	0.1929	0.0571	0.3063	0.0179	0.0005
	MCMC	1.5492	0.1918	0.0576	0.3062	0.0182	0.0005
40	MLE	1.6657	0.1834	0.0570	0.3035	0.0150	0.0004
	LINDLEY	1.6341	0.1721	0.0489	0.3021	0.0147	0.0004
	TIERNEY–KADANE	1.5612	0.1659	0.0426	0.3047	0.0152	0.0004
	MCMC	1.5595	0.1691	0.0441	0.3045	0.0154	0.0004
50	MLE	1.6501	0.1608	0.0426	0.3029	0.0134	0.0003
	LINDLEY	1.6254	0.1527	0.0378	0.3017	0.0133	0.0003
	TIERNEY–KADANE	1.5674	0.1482	0.0341	0.3039	0.0136	0.0003
	MCMC	1.5700	0.1503	0.0355	0.3034	0.0135	0.0003
70	MLE	1.6365	0.1336	0.0296	0.3019	0.0114	0.0002
	LINDLEY	1.6192	0.1288	0.0271	0.3011	0.0113	0.0002
	TIERNEY–KADANE	1.5779	0.1262	0.0251	0.3026	0.0115	0.0002
	MCMC	1.5790	0.1288	0.0258	0.3025	0.0115	0.0002
100	MLE	1.6257	0.1112	0.0201	0.3013	0.0093	0.0001
	LINDLEY	1.6138	0.1085	0.0189	0.3007	0.0092	0.0001
	TIERNEY–KADANE	1.5849	0.1070	0.0179	0.3018	0.0093	0.0001
	MCMC	1.5857	0.1071	0.0179	0.3018	0.0094	0.0001
150	MLE	1.6167	0.0889	0.0127	0.3008	0.0076	0.0001
	LINDLEY	1.6088	0.0873	0.0122	0.3004	0.0076	0.0001
	TIERNEY–KADANE	1.5897	0.0865	0.0118	0.3011	0.0076	0.0001
	MCMC	1.5891	0.0880	0.0121	0.3012	0.0078	0.0000
200	MLE	1.6123	0.0768	0.0093	0.3006	0.0065	0.0001
	LINDLEY	1.6064	0.0759	0.0091	0.3003	0.0065	0.0001
	TIERNEY–KADANE	1.5921	0.0753	0.0088	0.3008	0.0066	0.0001
	MCMC	1.5932	0.0761	0.0091	0.3009	0.0067	0.0000
350	MLE	1.6074	0.0586	0.0054	0.3004	0.0050	0.0001
	LINDLEY	1.6040	0.0582	0.0054	0.3002	0.0050	0.0001
	TIERNEY–KADANE	1.5958	0.0580	0.0053	0.3005	0.0050	0.0001
	MCMC	1.5969	0.0571	0.0051	0.3007	0.0050	0.0000
500	MLE	1.6056	0.0484	0.0037	0.3003	0.0041	0.00003
	LINDLEY	1.6032	0.0481	0.0037	0.3001	0.0041	0.00003
	TIERNEY–KADANE	1.5975	0.0479	0.0036	0.3004	0.0041	0.00003
	MCMC	1.5981	0.0477	0.0036	0.3003	0.0041	0.00000

**Table 4:** ML, appoximate Bayes and MCMC method estimates, MSE and bias values, for $\alpha = 0.9$ and $\lambda = 2.1$

*n*	Estımation methods	$\alpha$			$\lambda$		
		Average	Bias	MSE	Average	Bias	MSE
10	MLE	1.0807	0.2739	0.1561	2.3125	0.4364	0.3811
	LINDLEY	1.0247	0.2485	0.1164	1.8283	0.4141	0.2436
	TIERNEY–KADANE	0.9117	0.1831	0.0572	2.1824	0.3470	0.2147
	MCMC	0.9147	0.1832	0.0577	2.2010	0.3553	0.2325
20	MLE	0.9822	0.1625	0.0488	2.1999	0.2849	0.1453
	LINDLEY	0.9573	0.1566	0.0437	1.9783	0.2582	0.0983
	TIERNEY–KADANE	0.9098	0.1346	0.0305	2.1532	0.2587	0.1150
	MCMC	0.9063	0.1337	0.0295	2.1575	0.2586	0.1148
30	MLE	0.9514	0.1239	0.0264	2.1658	0.2254	0.0856
	LINDLEY	0.9347	0.1210	0.0246	2.0225	0.2094	0.0657
	TIERNEY–KADANE	0.9055	0.1095	0.0194	2.1390	0.2126	0.0744
	MCMC	0.9055	0.1093	0.0197	2.1307	0.2093	0.0734
40	MLE	0.9374	0.1039	0.0183	2.1529	0.1921	0.0630
	LINDLEY	0.9248	0.1022	0.0174	2.0462	0.1794	0.0502
	TIERNEY–KADANE	0.9038	0.0950	0.0146	2.1340	0.1837	0.0568
	MCMC	0.9026	0.0963	0.0149	2.1306	0.1833	0.0562
50	MLE	0.9298	0.0915	0.0139	2.1402	0.1718	0.0493
	LINDLEY	0.9196	0.0904	0.0134	2.0558	0.1635	0.0412
	TIERNEY–KADANE	0.9032	0.0853	0.0116	2.1260	0.1662	0.0454
	MCMC	0.9036	0.0846	0.0116	2.1222	0.1618	0.0426
70	MLE	0.9204	0.0760	0.0093	2.1236	0.1414	0.0325
	LINDLEY	0.9130	0.0754	0.0091	2.0641	0.1378	0.0292
	TIERNEY–KADANE	0.9017	0.0724	0.0082	2.1141	0.1383	0.0308
	MCMC	0.9021	0.0723	0.0084	2.1170	0.1379	0.0308
100	MLE	0.9136	0.0614	0.0062	2.1193	0.1182	0.0224
	LINDLEY	0.9083	0.0611	0.0060	2.0778	0.1150	0.0205
	TIERNEY–KADANE	0.9006	0.0595	0.0057	2.1130	0.1163	0.0216
	MCMC	0.9027	0.0599	0.0057	2.1126	0.1154	0.0215
150	MLE	0.9103	0.0509	0.0042	2.1111	0.0945	0.0143
	LINDLEY	0.9067	0.0507	0.0041	2.0837	0.0933	0.0136
	TIERNEY–KADANE	0.9017	0.0497	0.0039	2.1070	0.0936	0.0140
	MCMC	0.9027	0.0492	0.0038	2.1059	0.0929	0.0136
200	MLE	0.9074	0.0436	0.0030	2.1078	0.0819	0.0107
	LINDLEY	0.9048	0.0430	0.0034	2.0873	0.0810	0.0103
	TIERNEY–KADANE	0.9010	0.0428	0.0029	2.1048	0.0813	0.0105
	MCMC	0.9002	0.0427	0.0029	2.1066	0.0810	0.0105
350	MLE	0.9043	0.0324	0.0017	2.1056	0.0624	0.0062
	LINDLEY	0.9028	0.0323	0.0016	2.0939	0.0620	0.0060
	TIERNEY–KADANE	0.9007	0.0321	0.0016	2.1040	0.0621	0.0061
	MCMC	0.9007	0.0319	0.0016	2.1043	0.0620	0.0060
500	MLE	0.9031	0.0027	0.0011	2.1035	0.0519	0.0043
	LINDLEY	0.9021	0.0270	0.0011	2.0953	0.0517	0.0042
	TIERNEY–KADANE	0.9006	0.0268	0.0011	2.1023	0.0518	0.0042
	MCMC	0.9002	0.0266	0.0011	2.1028	0.0517	0.0042

And also for each sample size n ∈ {20,30,40,50,70,100,150}, 500 independent datasets were generated under the true parameter values $\alpha$ = 1.6 and $\lambda$ = 0.3, with each MLE followed by 250 bootstrap replications. The primary evaluation metrics are empirical coverage probabilities and average confidence interval lengths.

**Fig. 2:**
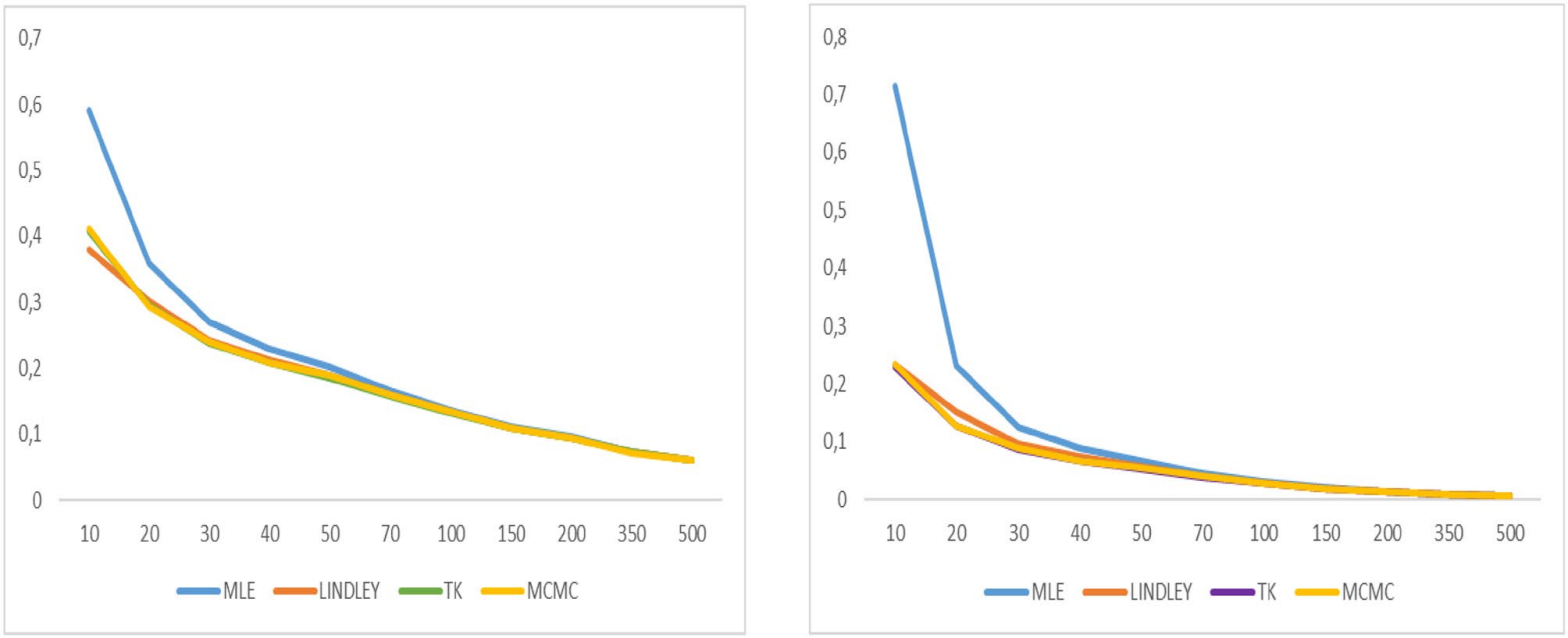
Graphs of bias value and MSE for α = 2.

**Fig. 3:**
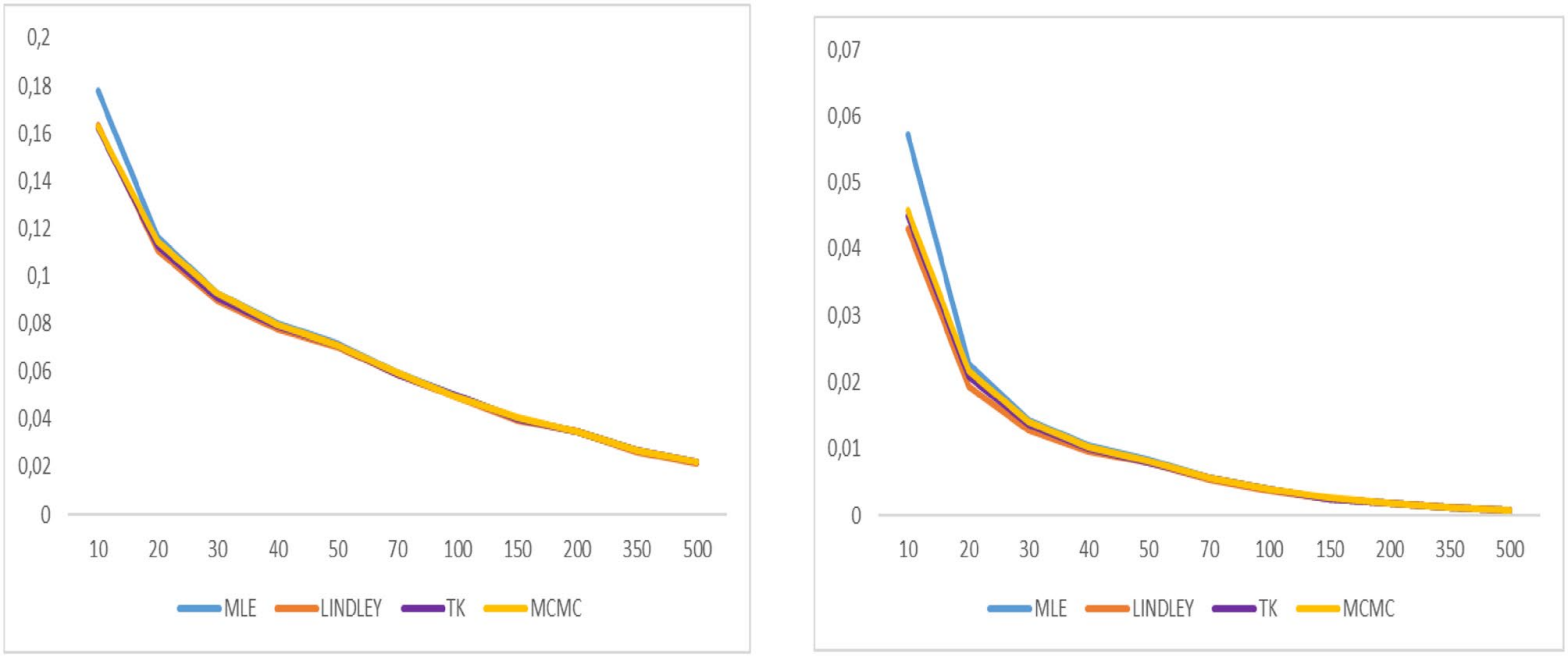
Graphs of bias value and MSE for λ = 2.

**Fig. 4:**
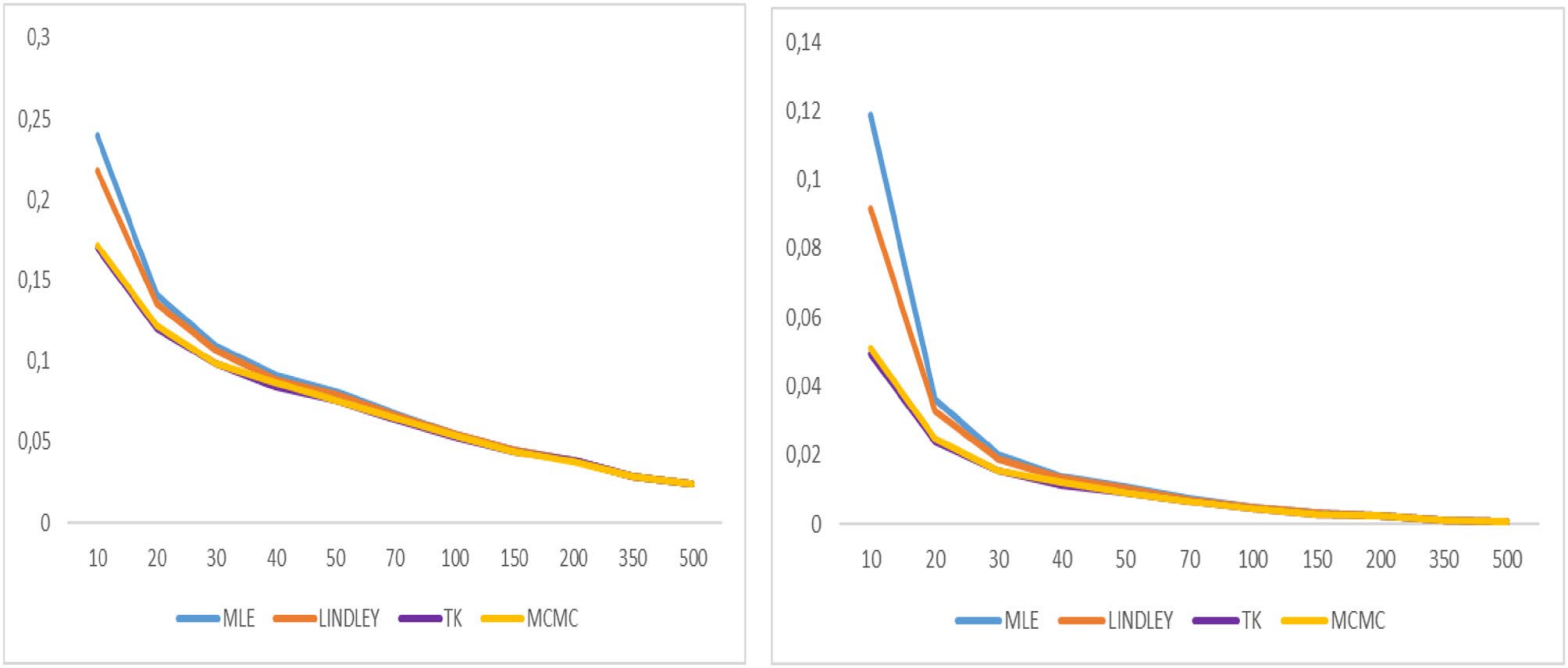
Graphs of bias value and MSE for α = 0.8.

**Fig. 5:**
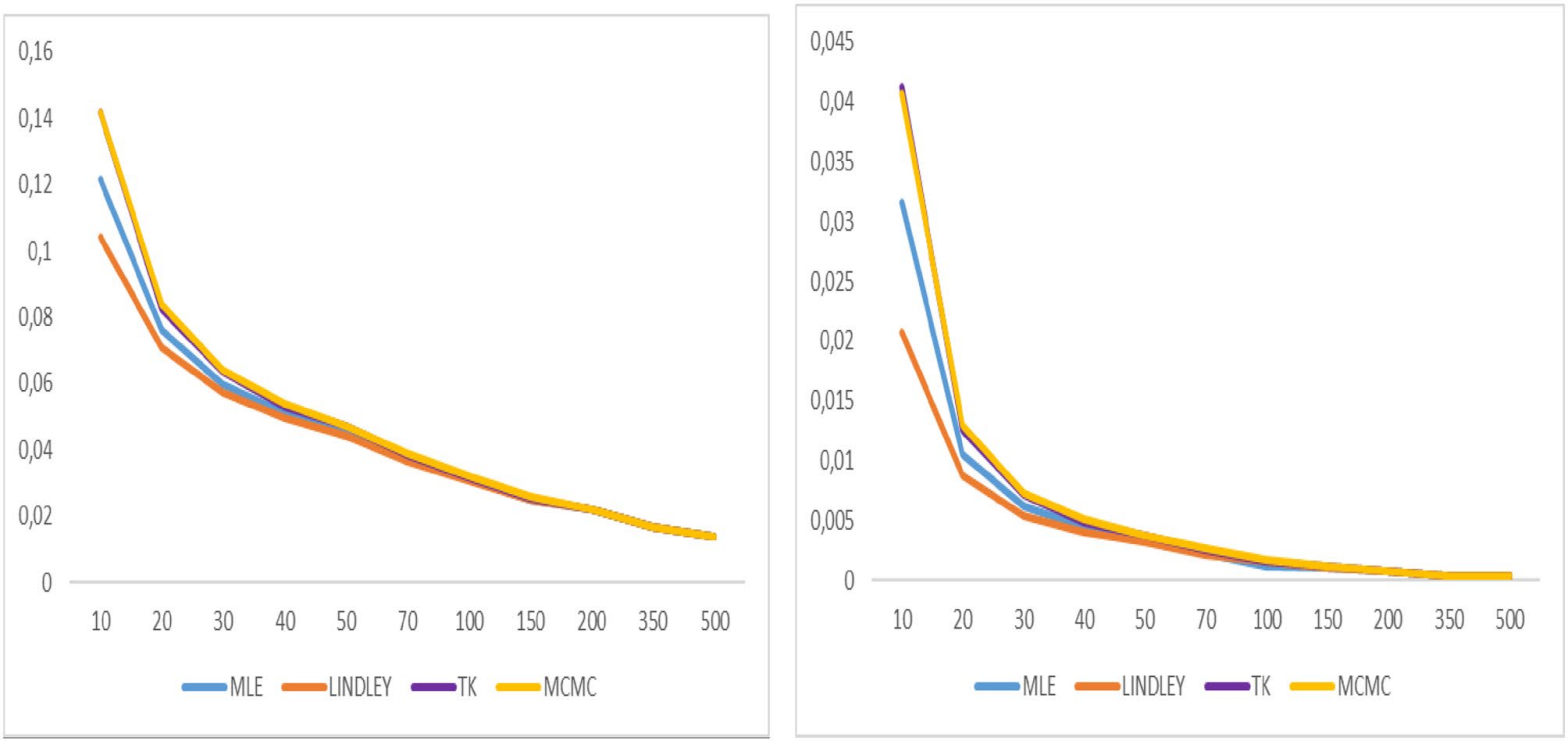
Graphs of bias value and MSE for λ = 0.5.

**Fig. 6:**
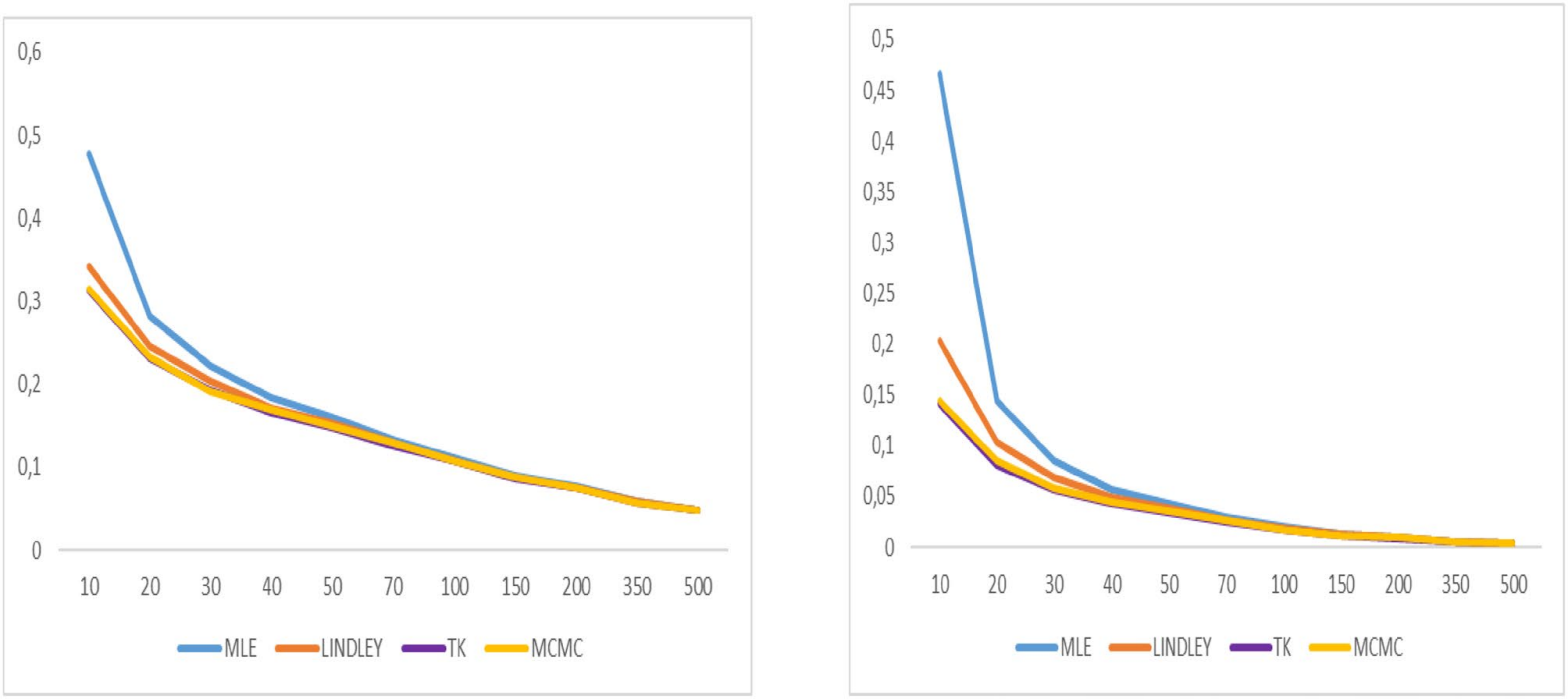
Graphs of bias value and MSE for $\alpha = 1.6$

**Fig. 7:**
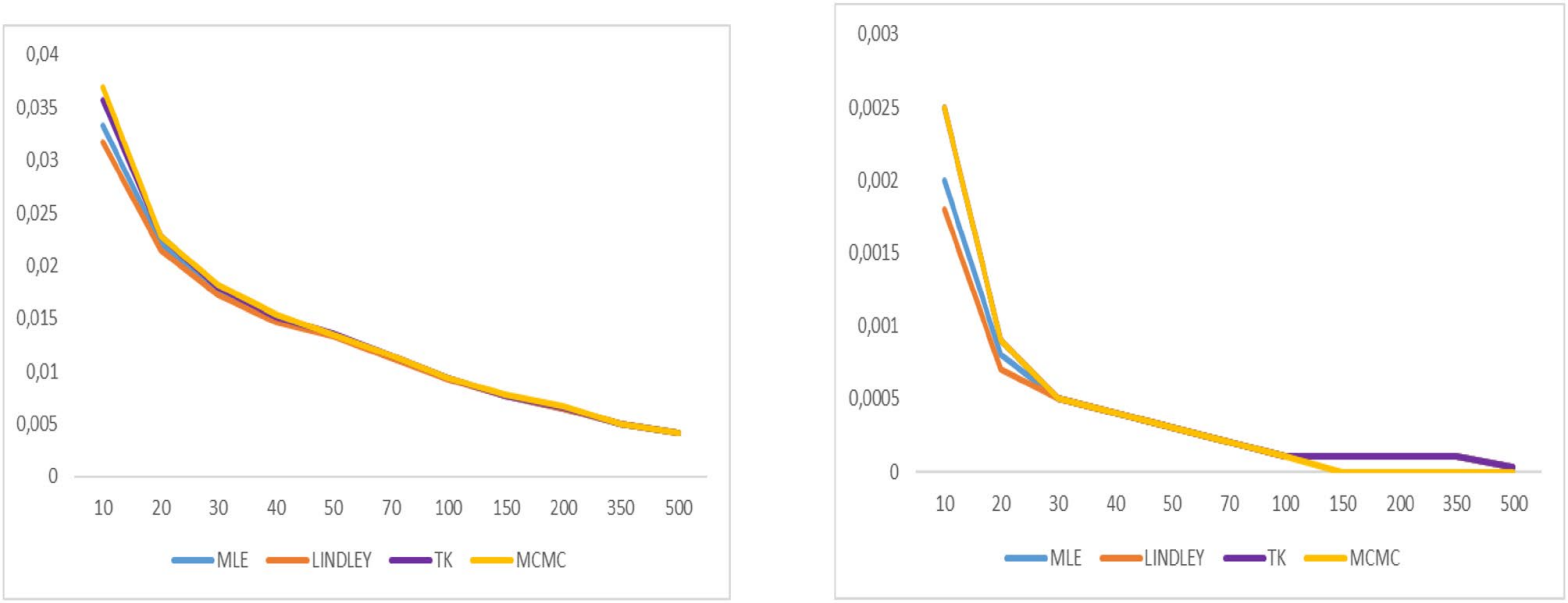
Graphs of bias value and MSE for λ = 0.3.

**Fig. 8:**
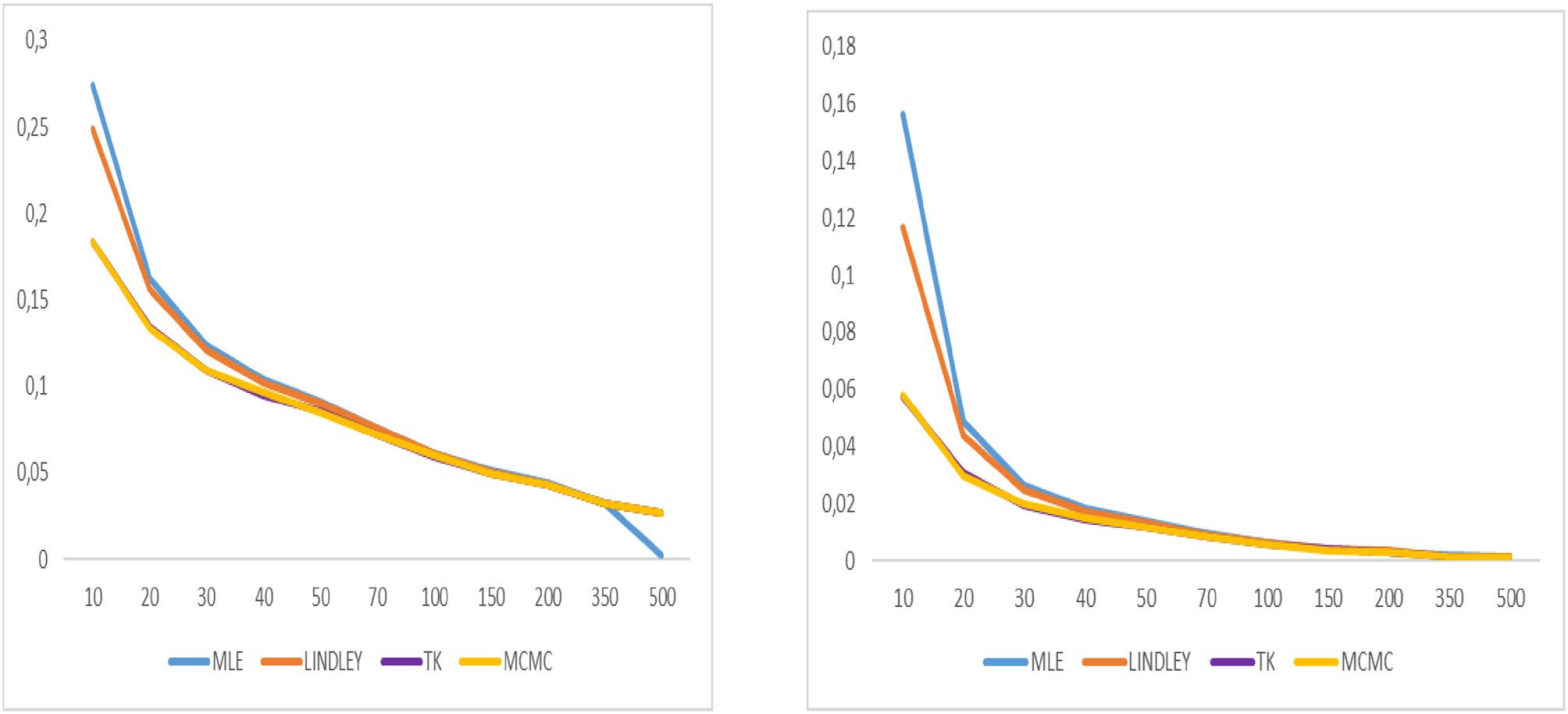
Graphs of bias value and MSE for α = 0.9.

**Fig. 9:**
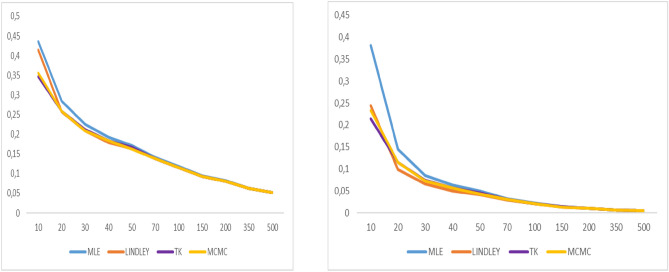
Graphs of bias value and MSE for λ = 2.1.

As shown in Tables 1, 2, 3 and 4, MSE and the bias values of all estimates are given for various $n$ values. The MCMC-based Bayes estimates outperform the ML estimates and the approximate Bayes estimates derived using Lindley and Tierney–Kadane. For ML and Bayes estimator methods, it is observed that when the $n$ values increase, the bias, MSEs of the estimates decrease to zero. Moreover, as indicated in Table 5, when the n values increase, the coverage probabilities attain the desired level, as expected. The coverage probabilities of the bootstrap confidence intervals are approximately 1 − α = 0.95 for various values of n. Figures 2, 3, 4, 5, 6, 7, 8 and 9 represent the comparison of biases and MSEs of the proposed estimates.

**Table 5:** Confidence average width and coverage probability for the bootstrap confidence interval of the parameter $\alpha$ and $\lambda$.

	$\alpha$				$\lambda$			
*n*	MLE coverage	MLE length	Percentile coverage	Percentile length	MLE coverage	MLE length	Percentile coverage	Percentile length
20	0.910	1.236	0.948	2.341	0.870	0.096	0.922	0.284
30	0.902	0.981	0.942	2.045	0.888	0.078	0.914	0.272
40	0.906	0.838	0.948	1.847	0.896	0.068	0.918	0.256
50	0.918	0.746	0.958	1.744	0.906	0.061	0.934	0.249
70	0.960	0.646	0.914	0.665	0.930	0.054	0.914	0.054
100	0.956	0.534	0.924	0.541	0.948	0.045	0.938	0.045
150	0.948	0.434	0.948	0.434	0.944	0.037	0.932	0.037

Although Lindley’s and Tierney–Kadane’s approximations offer computational advantages in Bayesian estimation, they inherently introduce certain limitations and potential biases. Lindley’s method, while analytically tractable and easy to implement, exhibits an approximation error of order O(n⁻^1^), making it more prone to bias, especially in small samples. In contrast, Tierney–Kadane’s method improves accuracy through a higher-order approximation O(n⁻^2^), but it requires more complex computations, such as evaluating second derivatives of the log-posterior. In our simulation results given in Tables 1, 2, 3 and 4, these differences are reflected in practice: Tierney–Kadane estimators tend to be closer to the MCMC benchmarks, whereas Lindley estimators demonstrate slightly greater deviation. This empirical finding confirms the theoretical trade-off between computational efficiency and estimation accuracy. Accordingly, we emphasize that the choice of approximation should consider both the available computational resources and the required precision for inference.

In this simulation study, two confidence interval (CI) methods show convergence toward the nominal 0.95 coverage level as the sample size increases, along with a steady reduction in average interval lengths approaching zero, reflecting the benefits of asymptotic efficiency. The MLE-based intervals tend to undercover in small sample scenarios**,** yielding coverage probabilities below the nominal level despite narrower interval lengths. The percentile bootstrap method also shows slight undercoverage for small n, though its performance improves with larger samples.

## Real data application of the Inverse Exponential Power (IEP) distribution to the COVID-19 pandemic case fatality rate

“Case Fatality Rate (CFR)” is defined as the ratio between “confirmed deaths” and “confirmed cases” as an important measure of the mortality risk of the COVID-19 Pandemic (Ourworldindata,^29^). In this section, as a novel statistical approach to the COVID-19 Pandemic “Case Fatality Rate (CFR)” indicator is developed using the Inverse Exponential Power (IEP) distribution. Also in this section, by fitting the IEP distribution to the COVID-19 Pandemic CFR data, the parameters of the IEP distribution are emprically estimated using MLE, Lindley, Tierney–Kadane, and MCMC methods. For this aim, COVID-19 Pandemic CFR data is taken from World Health Organization (WHO) regions as African Region (AFR), Eastern Mediterranean Region (EMR), European Region (EUR), Region of the Americas (AMR), South-East Asian Region (SEAR), Western Pacific Region (WPR), and also Organisation for Economic Co-Operation and Development (OECD) countries given in Table 6 (Ourworldindata,^30^; OECD Countries,^31^). The COVID-19 Pandemic CFR data belonging to the regions taken into the study are also given in Table 7.

**Table 6:** Countries of the WHO regions and OECD taken into the study (Ourworldindata,^30^; OECD Countries,^31^).

Regions	Countries
African Region (AFR)	Burundi, Seychelles, Mauritius, Benin, Gabon,Cape Verde, Togo, Central African Republic, South Sudan,Botswana, Ghana, Mozambique, Eritrea, Equatorial Guinea, Rwanda, Zambia, Nigeria, Guinea, Ethiopia, Mauritania,Cameroon, Sierra Leone, Kenya, Comoros, Burkina Faso, Angola, Guinea-Bissau, Eswatini, Tanzania, Lesotho, Madagascar, Uganda, Zimbabwe, Senegal, Mali, Namibia, South Africa, Chad, Algeria, Gambia, Malawi, Niger, Liberia
Eastern Mediterranean Region (EMR)	Qatar,United Arab Emirates, Bahrain, Kuwait, Jordan, Lebanon, Iraq, Saudi Arabia, Oman, Djibouti, Libya, Morocco, Iran, Pakistan, Tunisia, Afghanistan, Egypt, Somalia, Syria, Sudan, Yemen
European Region (EUR)	Iceland, Cyprus, Denmark, Israel, Luxembourg, Netherlands, Switzerland, Andorra, Austria, Norway, Monaco, France, Germany, Estonia, Portugal, San Marino, Ireland, Uzbekistan, Turkey, Finland, Greece, Slovenia, Tajikistan, Serbia, Belgium, Belarus, Malta, Lithuania, Italy, Latvia, Spain, Sweden, Georgia, United Kingdom, Montenegro, Albania, Slovakia, Kyrgyzstan, Azerbaijan, Kazakhstan, Croatia, Russia, Poland, Armenia, Moldova, Ukraine, Romania, Hungary, North Macedonia, Bulgaria, Bosnia and Herzegovina
Region of the Americas (AMR)	Dominica, Barbados, Dominican Republic, Saint Kitts and Nevis, Uruguay, Costa Rica, Cuba, Panama, Belize, Venezuela, United States, Canada, Chile, Grenada, Saint Vincent and the Grenadines, Argentina, Saint Lucia, Nicaragua, Guatemala, Antigua and Barbuda, Suriname, Guyana, Bolivia, Brazil, El Salvador, Bahamas, Colombia, Trinidad and Tobago, Jamaica, Honduras, Haiti, Paraguay, Ecuador, Mexico, Peru
South-East Asian Region (SEAR)	Bhutan, Maldives, Thailand, India, Nepal, Bangladesh, Indonesia, Sri Lanka, Myanmar
Western Pacific Region (WPR)	Nauru, Tuvalu, Brunei, Tonga, Singapore, Marshall Islands, Vanuatu, China, New Zealand, Palau, Samoa, Australia, Japan, Mongolia, Laos, Vietnam, Kiribati, Solomon Islands, Malaysia, Fiji, Papua New Guinea, Philippines, Cambodia
Organisation for Economic Co-Operation and Development (OECD)

**Table 7:** COVID-19 Pandemic CFR data belonging to the WHO regions and OECD taken into the study (Ourworldindata,^29^).

AFR REGION	0.338	0.341	0.582	0.627	0.646	0.734	0.735	0.751	0.848	0.852	0.961	1.011
	1.068	1.102	1.165	1.183	1.214	1.512	1.565	1.578	1.610	1.654	1.757	1.795
	1.835	1.841	1.906	1.964	2.047	2.086	2.115	2.150	2.214	2.241	2.383	2.519
	2.520	2.531	2.946	3.024	3.311	3.634						
EMR REGION	0.134	0.220	0.220	0.386	0.808	0.883	1.029	1.146	1.159	1.205	1.269	1.278
	1.922	1.939	2.551	3.534	4.812	4.979	5.508	7.885	18.075			
EUR REGION	0.206	0.257	0.261	0.261	0.267	0.318	0.331	0.371	0.378	0.397	0.431	0.455
	0.482	0.484	0.513	0.532	0.591	0.596	0.680	0.697	0.701	0.703	0.709	0.716
	0.716	0.730	0.733	0.737	0.756	0.872	0.909	0.925	0.927	1.057	1.079	1.134
	1.165	1.237	1.269	1.435	1.740	1.835	1.948	1.953	1.992	2.001	2.215	2.854
	2.955	4.057										
AMR REGION	0.470	0.566	0.657	0.696	0.735	0.761	0.765	0.826	0.972	1.060	1.090	1.131
	1.165	1.209	1.288	1.299	1.361	1.533	1.592	1.603	1.705	1.770	1.857	1.868
	2.096	2.216	2.241	2.292	2.307	2.345	2.507	2.713	3.371	4.380	4.901	
SEAR REGION	0.169	0.724	1.182	1.199	1.441	2.377	2.510	3.041				
WPR REGION	0.106	0.116	0.122	0.137	0.145	0.185	0.195	0.221	0.226	0.307	0.372	0.472
	0.708	0.726	1.282	1.430	1.597	2.200						
OECD	0,105	0,137	0,196	0,221	0,257	0,261	0,261	0,267	0,318	0,371	0,378	0.431
	0.455	0.482	0.532	0.596	0.680	0.697	0.701	0.716	0.733	0.737	0.756	0.761
	0.872	0.909	0.922	0.927	1.090	1.131	1.134	1.165	1.835	2.215	2.242	4.380

**Fig. 10:**
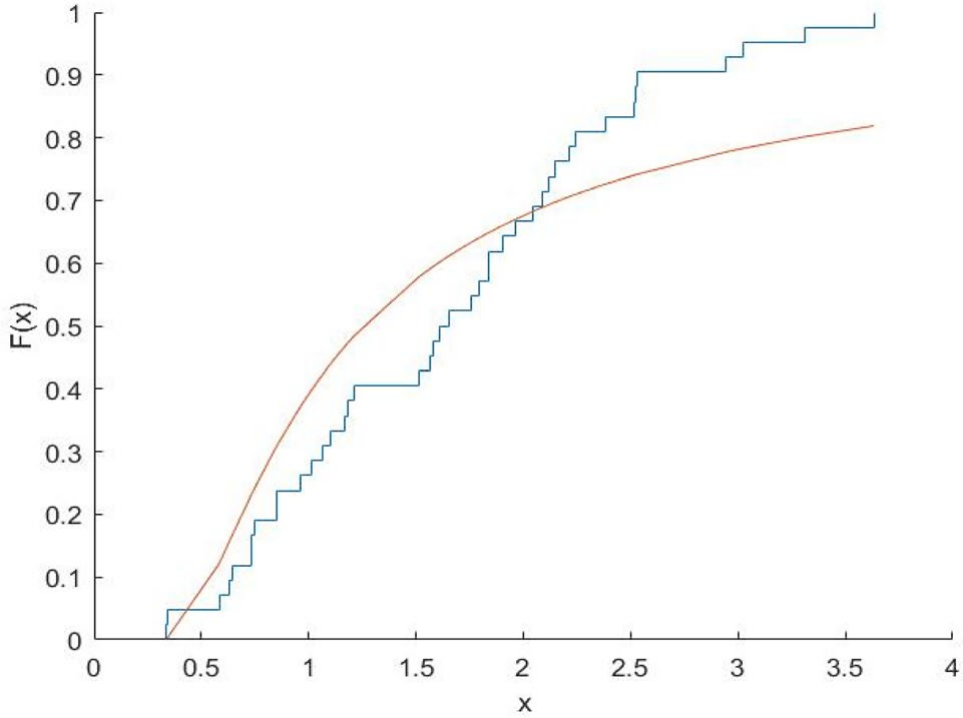
Plot Empricial CDF and MFE CDF for the Afr Region.

**Fig. 11:**
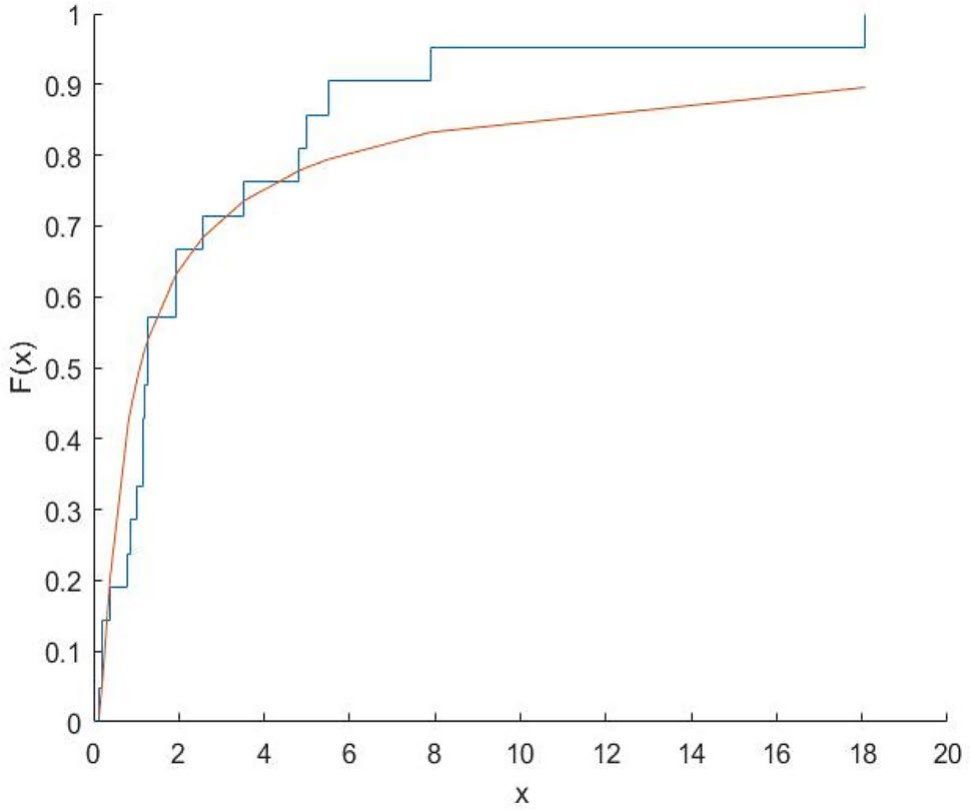
Plot Empricial CDF and MFE CDF for the Emr Region.

**Fig. 12:**
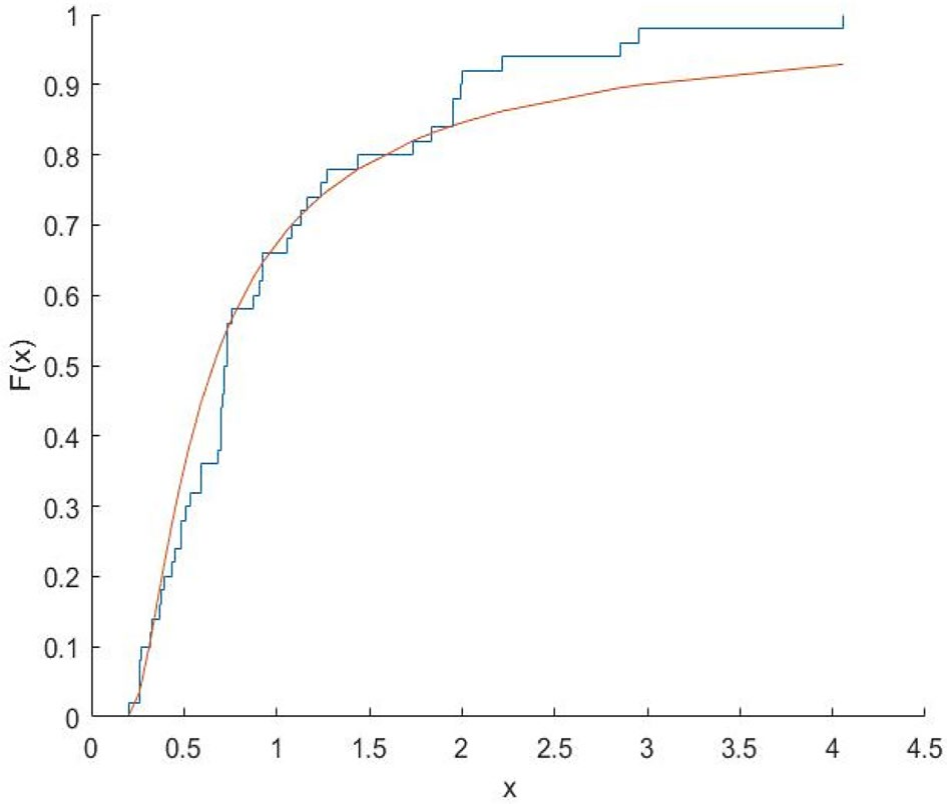
Plot Empricial CDF and MFE CDF for the Eur Region.

**Fig. 13:**
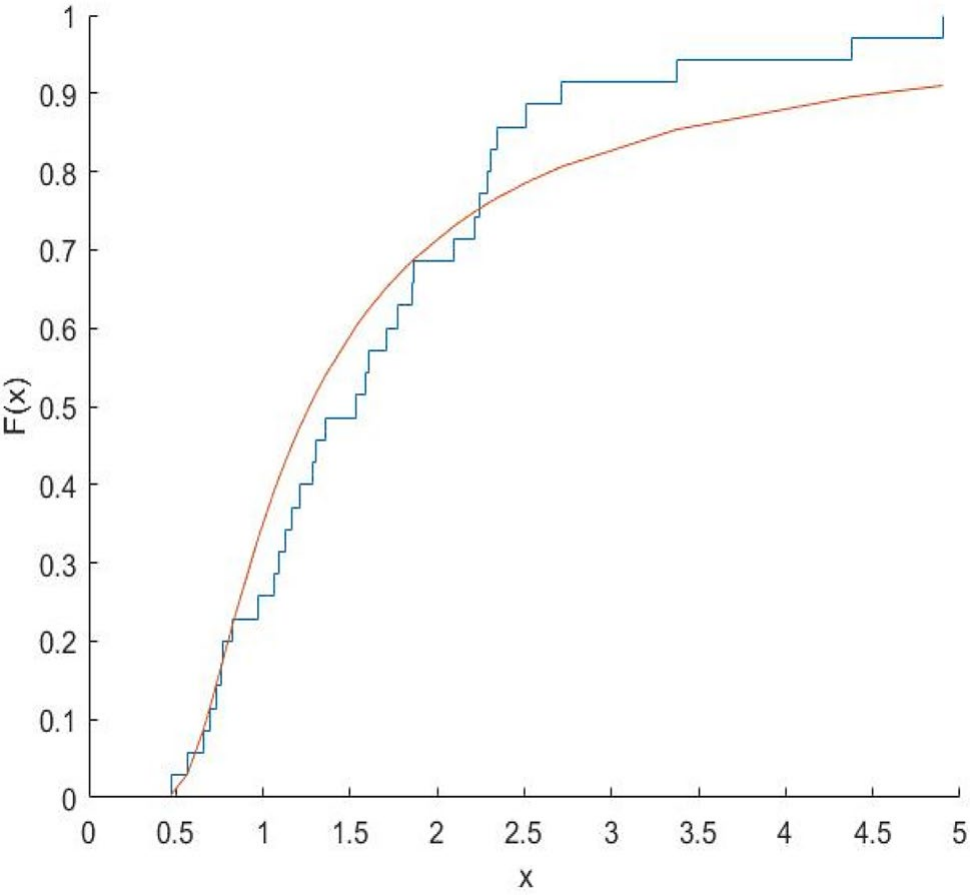
Plot Empricial CDF and MFE CDF for the Amr Region.

**Fig. 14:**
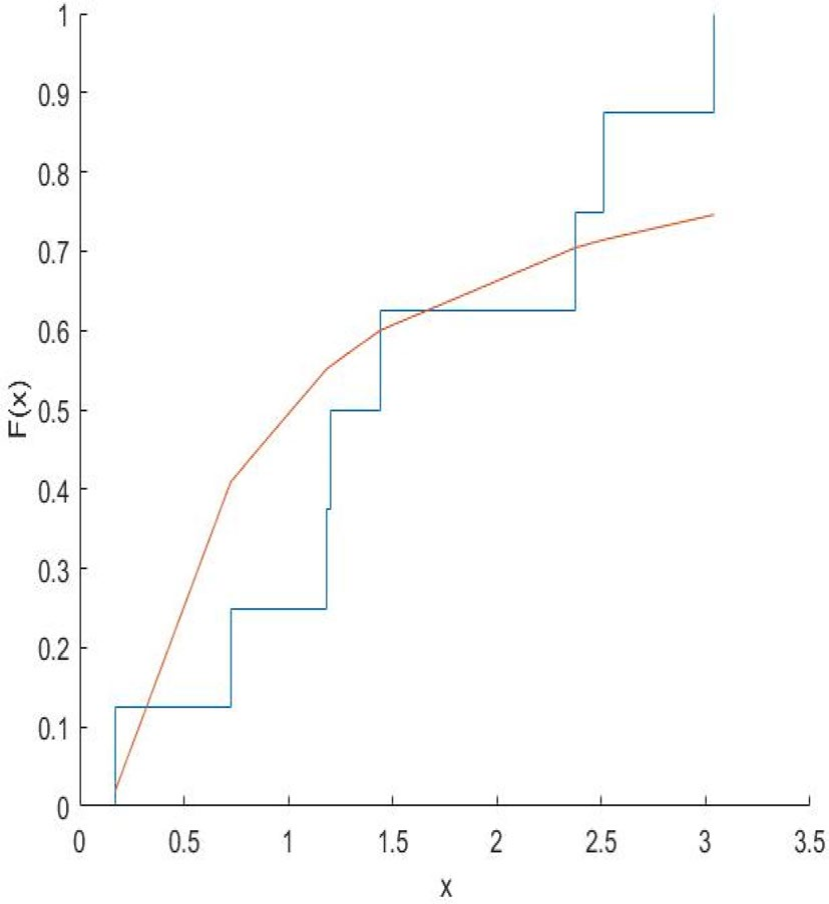
Plot Empricial CDF and MFE CDF for the Sear Region.

**Fig. 15:**
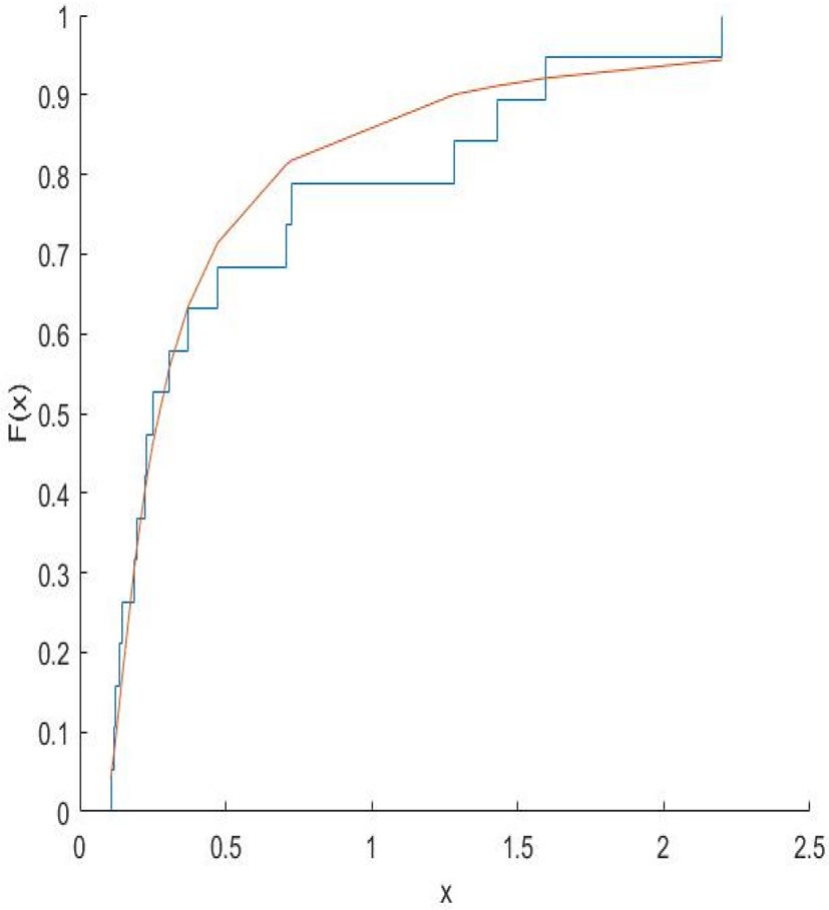
Plot Empricial CDF and MFE CDF for the Wpr Region.

**Fig. 16:**
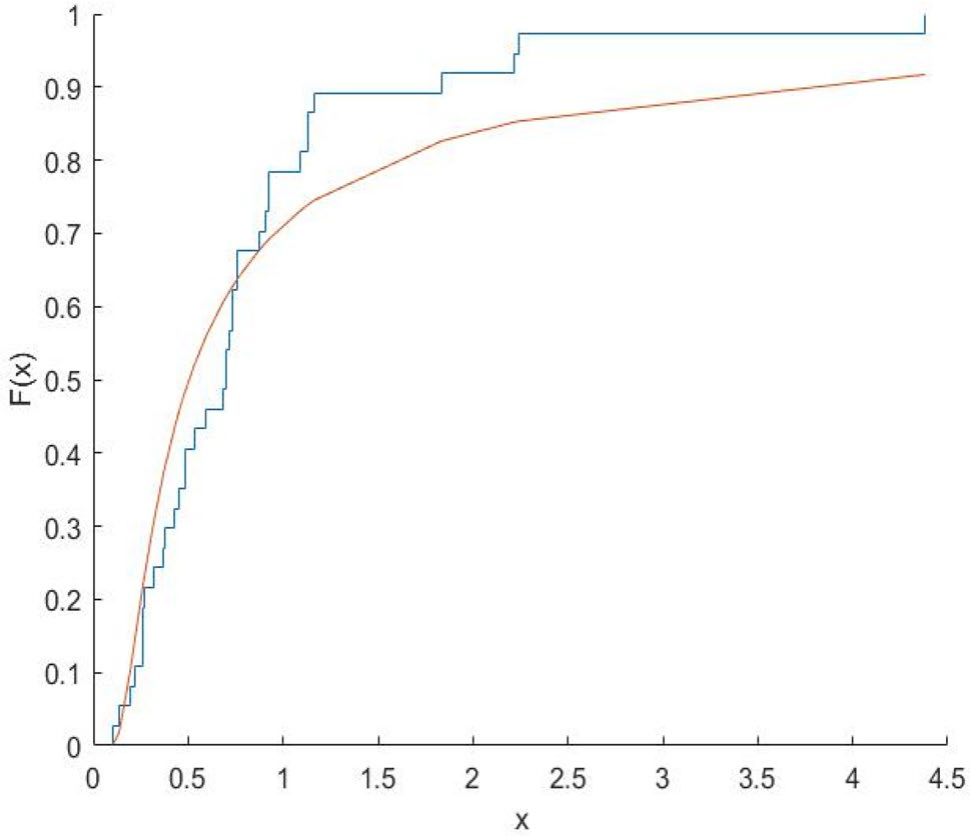
Plot Empricial CDF and MFE CDF for the OECD Region.

The Kolmogorov–Smirnov test statistics and the associated p-values are computed and shown in Table 8. Figures 10, 11, 12, 13, 14, 15 and 16 gives the comparison between empicical CDF and theoretical CDF of COVID-19 Pandemic CFR data belongs to different region for the proposed distribution.

**Table 8:** Kolmogorov–Smirnov Z and the corresponding *p*-values for the ML estimates.

WHO REGION/OECD	Estimates	Kolmogorov–Smirnov Z	*p*-values
AFR	$\hat{\alpha }_{MLE} = 0.9993,\hat{\lambda }_{MLE} = 0.6609$	0.1810	0.1274
EMR	$\hat{\alpha }_{MLE} = 3.3000,\hat{\lambda }_{MLE} = 1.2000$	0.2291	0.2203
EUR	$\hat{\alpha }_{MLE} = \, 1.1008,\hat{\lambda }_{MLE} = 0.3674$	0.1561	0.1749
AMR	$\hat{\alpha }_{MLE} = 1.3035,\hat{\lambda }_{MLE} = 0.7742$	0.1326	0.5699
SEAR	$\hat{\alpha }_{MLE} = 0.6341,\hat{\lambda }_{MLE} = 0.3566$	0.3008	0.4640
WPR	$\hat{\alpha }_{MLE} = 1.1681,\hat{\lambda }_{MLE} = 0.1444$	0.1287	0.9114
OECD	$\hat{\alpha }_{MLE} = 0.8557,\hat{\lambda }_{MLE} = 0.2388$	0.1465	0.4051

In Table 9, we obtained the proposed estimates of the model parameters using the data set for different regions.

**Table 9:** ML and approximate Bayes estimates computed with Lindley aproximation, Tierney–Kadane aproximation and MCMC method for $\alpha$ and $\lambda$ parameters.

WHO REGION/OECD	Estimators	$\hat{\alpha }$	$\hat{\lambda }$
AFR	MLE	0.9993	0.6609
	LINDLEY	0.9813	0.6532
	TIERNEY–KADANE	0.9645	0.6695
	MCMC	0.9589	0.6711
EMR	MLE	0.5754	0.3572
	LINDLEY	0.5602	0.3545
	TIERNEY–KADANE	0.5422	0.3941
	MCMC	0.5446	0.3951
EUR	MLE	1.1008	0.3674
	LINDLEY	1.0897	0.3655
	TIERNEY–KADANE	1.0629	0.3709
	MCMC	1.0613	0.3716
AMR	MLE	1.3035	0.7742
	LINDLEY	1.2788	0.7651
	TIERNEY–KADANE	1.2349	0.7799
	MCMC	1.2371	0.7780
SEAR	MLE	0.6341	0.3566
	LINDLEY	0.5904	0.3481
	TIERNEY–KADANE	0.5441	0.4591
	MCMC	0.5345	0.4903
WPR	MLE	1.1681	0.1444
	LINDLEY	1.1481	0.1433
	TIERNEY–KADANE	1.0502	0.1480
	MCMC	0.9454	0.1526
OECD	MLE	0.8557	0.2388
	LINDLEY	0.8418	0.2379
	TIERNEY–KADANE	0.8220	0.2450
	MCMC	0.8092	0.2453

## Conclusion

In this study, we investigated the maximum likelihood (ML) and approximate Bayesian estimation methods for the parameters of the Inverse Exponential Power (IEP) distribution under complete sampling. While the ML estimators were obtained via the Newton–Raphson algorithm, Bayesian estimators were derived using Lindley’s approximation, Tierney–Kadane expansion, and the MCMC method, all under a squared-error loss function.

Monte Carlo simulation results revealed that, although all estimators performed satisfactorily, the MCMC-based Bayesian estimates consistently outperformed the others in terms of bias and mean squared error, particularly for small and moderate sample sizes. Additionally, the width of bootstrap confidence intervals narrowed as sample size increased, and coverage probabilities approached the nominal 95% level, indicating reliability of the proposed procedures.

Also in this study, a novel statistical approach to the COVID-19 Pandemic “Case Fatality Rate (CFR)” indicator is developed using the Inverse Exponential Power (IEP) distribution. By fitting the IEP distribution to the COVID-19 Pandemic CFR data, the parameters of the IEP distribution are emprically estimated using MLE, Lindley, Tierney–Kadane, and MCMC methods.

This research not only contributes to the literature by providing the first Bayesian treatment of the IEP distribution but also offers a comparative framework for evaluating approximation techniques in Bayesian inference. Future work may focus on developing analogous estimation techniques for discrete versions of the IEP model or for other health indicator distributions, especially in applications involving discrete data such as count data in medical and epidemiological studies.

Looking ahead, a future study could build upon the framework proposed by Xu et al.^32^, integrating adaptive sampling and ensemble-based federated deep learning for decentralized COVID-19 data analysis. This approach would enable privacy-preserving model training across institutions, improve robustness through ensemble methods, and facilitate dynamic learning via Bayesian deep networks. Such advancements could significantly enhance real-time health monitoring and support data-driven decision-making in global public health.

## Supplementary Information

Supplementary Information.

## Data Availability

Case fatality rate of COVID-19 dataset analysed during this study are included in Our World in Data [30]. (https://ourworldindata.org/explorers/covid?tab=table&Metric=Case+fatality+rate&Interval=Cumulative&Relative+to+population=true).
