# Supplementary material for: Bayesian estimation of the inverse Exponential Power distribution for COVID-19 case fatality analysis under SDG 3
Source: Sci Rep. 2025 Dec 1;15:43202. doi: 10.1038/s41598-025-27264-7 (PMC12680629; doi:10.1038/s41598-025-27264-7)
Supplement: Supplementary file 1 — Supplementary Information. [file 41598_2025_27264_MOESM1_ESM.docx]

Manuscript Title: **Bayesian Estimation of the Inverse Exponential Power Distribution for COVID-19 Case Fatality Analysis under SDG 3**

**Appendix**

 A1

 A2

*$L_{21}=L_{12}$* A3

$\begin{matrix} L_{22}=\frac{\partial^{2}L}{\partial\lambda^{2}}=-\frac{n\alpha}{\lambda^{2}}+\frac{\alpha\left(\alpha-1\right)}{\lambda^{2}}\sum_{i=1}^{n} \left(\frac{\lambda}{x_{i}}\right)^{\alpha} \\ -\frac{\alpha}{\lambda^{2}}\sum_{i=1}^{n} \exp\left(\frac{\lambda}{x_{i}}\right)^{\alpha}\left(\frac{\lambda}{x_{i}}\right)^{\alpha}\left(\alpha\left(\frac{\lambda}{x_{i}}\right)^{\alpha}+\alpha-1\right) \end{matrix}$ A4

$\begin{matrix} L_{111}=\frac{\partial^{3}L}{\partial\alpha^{3}}=\frac{2n}{\alpha^{3}}+\sum_{i=1}^{n} \left(\frac{\lambda}{x_{i}}\right)^{\alpha}\log\left(\frac{\lambda}{x_{i}}\right)^{3} \\ -\sum_{i=1}^{n} \exp\left(\frac{\lambda}{x_{i}}\right)^{\alpha}\left(\frac{\lambda}{x_{i}}\right)^{\alpha}\log\left(\frac{\lambda}{x_{i}}\right)^{3}\left[1+3\left(\frac{\lambda}{x_{i}}\right)^{\alpha}+\left(\frac{\lambda}{x_{i}}\right)^{2\alpha}\right] \end{matrix}$ A5

$\begin{matrix} L_{112}=\frac{\partial^{3}L}{\partial\alpha^{2}\partial\lambda}=\sum_{i=1}^{n} \left(\frac{\lambda}{x_{i}}\right)^{\alpha}\log\left(\frac{\lambda}{x_{i}}\right)\left[\frac{\alpha}{\lambda}\log\left(\frac{\lambda}{x_{i}}\right)+\frac{2}{\lambda}\right] \\ -\frac{\alpha}{\lambda}\sum_{i=1}^{n} \exp\left(\frac{\lambda}{x_{i}}\right)^{\alpha}\left(\frac{\lambda}{x_{i}}\right)^{\alpha}\log\left(\frac{\lambda}{x_{i}}\right)^{2}\left[1+3\left(\frac{\lambda}{x_{i}}\right)^{\alpha}+\left(\frac{\lambda}{x_{i}}\right)^{2\alpha}\right] \\ -\frac{2}{\lambda}\sum_{i=1}^{n} \exp\left(\frac{\lambda}{x_{i}}\right)^{\alpha}\left(\frac{\lambda}{x_{i}}\right)^{\alpha}\log\left(\frac{\lambda}{x_{i}}\right)\left[1+\left(\frac{\lambda}{x_{i}}\right)^{\alpha}\right] \end{matrix}$ A6

*$L_{121}=L_{211}=L_{112}$* A7

$\begin{matrix} L_{122}=\frac{\partial^{3}L}{\partial\alpha\partial\lambda^{2}}=-\frac{n}{\lambda^{2}}+\frac{\alpha^{2}-\alpha}{\lambda^{2}}\sum_{i=1}^{n} \left(\frac{\lambda}{x_{i}}\right)^{\alpha}\log\left(\frac{\lambda}{x_{i}}\right)+\frac{2\alpha-1}{\lambda^{2}}\sum_{i=1}^{n} \left(\frac{\lambda}{x_{i}}\right)^{\alpha} \\ -\frac{\alpha^{2}}{\lambda^{2}}\sum_{i=1}^{n} \exp\left(\frac{\lambda}{x_{i}}\right)^{\alpha}\left(\frac{\lambda}{x_{i}}\right)^{\alpha}\log\left(\frac{\lambda}{x_{i}}\right)\left[1+3\left(\frac{\lambda}{x_{i}}\right)^{\alpha}+\left(\frac{\lambda}{x_{i}}\right)^{2\alpha}\right] \\ +\frac{\alpha}{\lambda^{2}}\sum_{i=1}^{n} \exp\left(\frac{\lambda}{x_{i}}\right)^{\alpha}\left(\frac{\lambda}{x_{i}}\right)^{\alpha}\log\left(\frac{\lambda}{x_{i}}\right)\left[1+\left(\frac{\lambda}{x_{i}}\right)^{\alpha}\right] \\ -\frac{1}{\lambda^{2}}\sum_{i=1}^{n} \exp\left(\frac{\lambda}{x_{i}}\right)^{\alpha}\left(\frac{\lambda}{x_{i}}\right)^{\alpha}\left[2\alpha\left(1+\left(\frac{\lambda}{x_{i}}\right)^{\alpha}\right)-1\right] \end{matrix}$ A8

*$L_{221}=L_{212}=L_{122}$* A9

$\begin{matrix} L_{222}=\frac{\partial^{3}L}{\partial\lambda^{3}}=\frac{2n\alpha}{\lambda^{3}}+\frac{\alpha\left(\alpha^{2}-3\alpha+2\right)}{\lambda^{3}}\sum_{i=1}^{n} \left(\frac{\lambda}{x_{i}}\right)^{\alpha} \\ +\frac{3\alpha^{2}}{\lambda^{3}}\sum_{i=1}^{n} \exp\left(\frac{\lambda}{x_{i}}\right)^{\alpha}\left(\frac{\lambda}{x_{i}}\right)^{\alpha}\left(1+\left(\frac{\lambda}{x_{i}}\right)^{\alpha}\right) \\ -\frac{\alpha^{3}}{\lambda^{3}}\sum_{i=1}^{n} \exp\left(\frac{\lambda}{x_{i}}\right)^{\alpha}\left(\frac{\lambda}{x_{i}}\right)^{\alpha}\left[1+3\left(\frac{\lambda}{x_{i}}\right)^{\alpha}+\left(\frac{\lambda}{x_{i}}\right)^{2\alpha}\right] \\ -\frac{2\alpha}{\lambda^{3}}\sum_{i=1}^{n} \exp\left(\frac{\lambda}{x_{i}}\right)^{\alpha}\left(\frac{\lambda}{x_{i}}\right)^{\alpha} \end{matrix}$ A10
